# The mesencephalic locomotor region recruits V2a reticulospinal neurons to drive forward locomotion in larval zebrafish

**DOI:** 10.1038/s41593-023-01418-0

**Published:** 2023-09-04

**Authors:** Martin Carbo-Tano, Mathilde Lapoix, Xinyu Jia, Olivier Thouvenin, Marco Pascucci, François Auclair, Feng B. Quan, Shahad Albadri, Vernie Aguda, Younes Farouj, Elizabeth M. C. Hillman, Ruben Portugues, Filippo Del Bene, Tod R. Thiele, Réjean Dubuc, Claire Wyart

**Affiliations:** 1 Sorbonne Université, Paris Brain Institute (Institut du Cerveau, ICM), Institut National de la Santé et de la Recherche Médicale U1127, Centre National de la Recherche Scientifique Unité Mixte de Recherche 7225, Assistance Publique–Hôpitaux de Paris, Campus Hospitalier Pitié-Salpêtrière, Paris, France; 2 Institut Langevin, École Supérieure de Physique et de Chimie Industrielles de la Ville de Paris, Paris Sciences et Lettres, Centre National de la Recherche Scientifique, Paris, France; 3 Université Paris-Saclay, Commissariat à l’Énergie Atomique et aux Énergies Alternatives, Centre National de la Recherche Scientifique, NeuroSpin, Baobab, Centre d’études de Saclay, Gif-sur-Yvette, France; 4 The American University of Paris, Paris, France; 5 Département de Neurosciences, Faculté de Médecine, Université de Montréal, Montréal, Quebec Canada; 6 Sorbonne Université, Institut National de la Santé et de la Recherche Médicale, Centre National de la Recherche Scientifique, Institut de la Vision, Paris, France; 7 Department of Biological Sciences, University of Toronto Scarborough, Toronto, Ontario Canada; 8 Institute of Neuroscience, Technical University of Munich, Munich, Germany; 9 Laboratory for Functional Optical Imaging, Mortimer B. Zuckerman Mind Brain Behavior Institute, Columbia University, New York, NY USA; 10 Department of Biomedical Engineering, Columbia University, New York, NY USA; 11 Kavli Institute for Brain Science, Columbia University, New York, NY USA; 12 Munich Cluster of Systems Neurology (SyNergy), Munich, Germany; 13 Groupe de Recherche en Activité Physique Adaptée, Department of Exercise Science, Université du Québec à Montréal, Montréal, Quebec Canada

**Keywords:** Motor control, Neural circuits

## Abstract

The mesencephalic locomotor region (MLR) is a brain stem area whose stimulation triggers graded forward locomotion. How MLR neurons recruit downstream *vsx2*^+^ (V2a) reticulospinal neurons (RSNs) is poorly understood. Here, to overcome this challenge, we uncovered the locus of MLR in transparent larval zebrafish and show that the MLR locus is distinct from the nucleus of the medial longitudinal fasciculus. MLR stimulations reliably elicit forward locomotion of controlled duration and frequency. MLR neurons recruit V2a RSNs via projections onto somata in pontine and retropontine areas, and onto dendrites in the medulla. High-speed volumetric imaging of neuronal activity reveals that strongly MLR-coupled RSNs are active for steering or forward swimming, whereas weakly MLR-coupled medullary RSNs encode the duration and frequency of the forward component. Our study demonstrates how MLR neurons recruit specific V2a RSNs to control the kinematics of forward locomotion and suggests conservation of the motor functions of V2a RSNs across vertebrates.

## Abstract (web summary)

Carbo-Tano and colleagues investigate the mesencephalic locomotor region in larval zebrafish and its role in triggering forward locomotion by activating specific sets of hindbrain V2a reticulospinal neurons.

## Main

Locomotion is essential for animals to move in their environment. It can be sensory-evoked to avoid a threatening stimulus during escape responses, or used for navigation to explore their surroundings when searching for resources. Motor circuits in vertebrate species are conserved at the anatomical, physiological and molecular levels^1^. The production of locomotor movements relies on the recruitment of command neurons in the brain stem, referred to as reticulospinal neurons (RSNs) and located in the reticular formation. RSNs integrate synaptic inputs from higher brain areas and from the periphery; in turn, they instruct the spinal circuits that produce locomotor movements^1–3^. RSNs have a crucial role in starting, maintaining and stopping locomotion^4–6^, as well as in adjusting posture and steering^7^.

A critical region involved in the production of locomotion upstream of the reticular formation is the mesencephalic locomotor region (MLR), first identified in cats^8^. The MLR has since been defined in many other vertebrate species, including lampreys, salamanders, rats, mice, rabbits, guinea pigs, pigs and monkeys^9^. Electrical stimulation of the MLR triggers forward locomotion in a graded fashion as a function of stimulation intensity^10^. Conservation of MLR properties across vertebrate species suggests that this brain stem structure is essential to vertebrate locomotion.

Anatomical studies indicate that the MLR is localized in the vicinity of the mesopontine cholinergic nuclei^10^ and corresponds in mammals to the pedunculopontine nucleus^11^ (PPN) and the cuneiform nucleus^8^ (CnF). In all vertebrate species investigated, the MLR induces locomotion via the activation of RSNs^12–15^. However, the way the MLR recruits RSNs to elicit forward locomotion has not yet been resolved due to the difficulty in accessing neurons scattered across the reticular formation in vivo. An important subclass of RSNs acting as command neurons is defined by the expression of the transcription factor *vsx2* (referred subsequently to as ‘V2a’) RSNs^16,17^. V2a RSNs are necessary for the initiation of locomotion^18^. Recent studies in fish and mice highlighted the role of pontine (P), retropontine (RP) and rostral medullary V2a RSNs in steering^19–21^ and stopping locomotion^22^. Yet, whether V2a RSNs are recruited by the MLR to trigger forward motion at specific locomotor frequencies is unknown.

In this study, we demonstrate how MLR neurons differentially project and recruit a subset of genetically identified V2a RSNs in the P and RP region and medulla^23^ to control the kinematics of forward locomotion. We first identify anatomically and functionally the MLR locus in larval zebrafish in the prepontine (PP) region. Whole-brain calcium imaging during forward optomotor response revealed that MLR neurons were recruited during spontaneous and visually evoked locomotion. We describe that MLR neurons project onto the soma of P and RP V2a RSNs while they project on the dendrites of medial V2a RSNs in the medulla. We combine behavioral recordings and population calcium imaging on single-pulsed MLR stimulation and find that, consistent with our anatomical investigations, P and RP V2a RSNs are strongly coupled to the MLR while medial V2a RSNs in the caudal medulla are weakly coupled. In contrast, neurons from the nucleus medial longitudinal fasciculus (nMLF) are not recruited on MLR stimulation. High-speed volumetric imaging of the entire brain stem V2a population during spontaneous locomotion reveals that a distributed network of medial V2a RSNs in the P and RP area, and caudal medulla, encode forward locomotion. In contrast, V2a RSNs in the rostral medulla and caudal RP region are organized in two clusters specialized for steering. Upon MLR stimulation effectively triggering locomotion, we discover that a subset of medial V2a RSNs in the medulla are recruited to sustain and control the speed of forward locomotion.

## Results

### Functional identification of the MLR in larval zebrafish

**Fig. 1:**
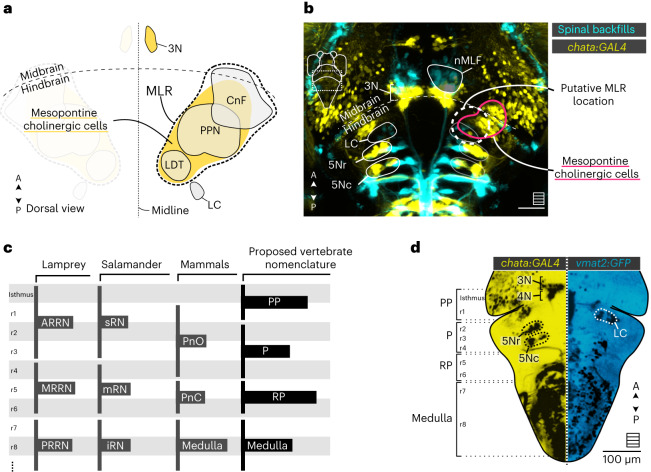
The putative location of the MLR in zebrafish inferred from previous studies in other vertebrates. **a**, Schematic illustration of the MLR location in vertebrates (dashed line) in relation to the mesopontine cholinergic cells (yellow). The structures classically defined as part of the MLR are the PPN, CnF and LDT. The characteristics of the structures were taken from the Allen Mouse Brain Atlas (https://atlas.brain-map.org). **b**, Schematic of the larval zebrafish brain depicting the probable location of the MLR (dashed white line, dorsal view) in relation to the LC and the mesopontine cholinergic cells labeled in the *Tg(chata:GAL4)* transgenic line. The hindbrain RSNs and the nMLF were labeled using spinal backfills. Scale bar, 50 µm. **c**, Scheme comparing the nomenclature and boundaries of the brain stem areas in different vertebrate classes. **d**, Applied nomenclature in the larval zebrafish brain stem. The anatomical landmarks were defined based on the projecting neurons labeled using spinal backfills (not shown) and the expression patterns of the *Tg(chata:GAL4)* (yellow) and *Tg(vmat2:GFP)* (cyan) transgenic lines. The PP region contains the isthmus and r1. The trochlear nucleus (4N) lies in the rostral border of the isthmus; among other structures, r1 contains the LC. The P region is defined rostrally by r2, containing the rostral trigeminal motor nucleus (5Nr), the caudal part of the trigeminal motor nucleus (5Nc) and caudally by rhombomere 4 (r4), including the Mauthner cell (M cell). The RP region consists of rhombomere 5 (r5) and rhombomere 6 (r6) containing the abducens (6N) and facial nucleus (7N), respectively. The medulla is delimited rostrally by rhombomere 7 (r7) and caudally by the rostral spinal cord. Under this nomenclature, the oculomotor nucleus (3N) is a midbrain structure, but the nMLF should be considered as a diencephalic structure. All images were taken from the Web interface of mapzebrain (https://fishatlas.neuro.mpg.de/). A, anterior; P, posterior.

Previous studies in fish reported that electrical stimulation of the dorsocaudal tegmentum in the midbrain that contains the nMLF induced locomotion^24–26^, leading some authors to suggest that this region corresponds to the MLR^24,27,28^. However, the following features of the nMLF argue against it being the MLR: (1) the nMLF is adjacent to the oculomotor nuclei (N3) and not to the mesopontine cholinergic nuclei where the MLR has been identified in all other vertebrates^9^ (Fig. 1a,b); and (2) nMLF neurons directly project to the spinal cord^29^ instead of mainly projecting onto RSNs. To identify the MLR locus of larval zebrafish, we therefore investigated using electrical stimulations with a monopolar tungsten microelectrode which loci of the mesopontine region would produce forward locomotion (Fig. 1b–d).

**Fig. 2:**
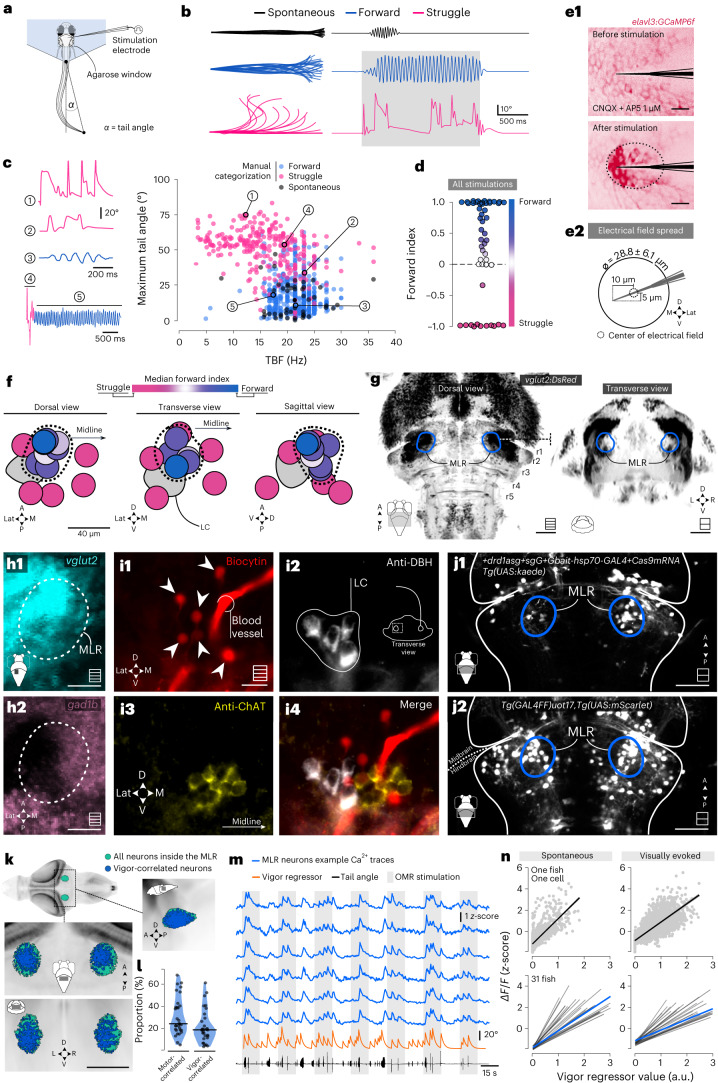
Anatomical and functional description of the MLR in larval zebrafish reveals neurons triggering forward locomotion and encoding vigor. **a**, Schematic illustration of the behavioral experiments with MLR stimulations. **b**, Typical behavioral responses shown as superimposed images of the larval zebrafish tail (left) and corresponding tail angle trace (right) of a spontaneous swim episode (black) and electrically evoked episodes corresponding to a forward swim (blue) or to a struggle (magenta). The gray bar indicates the stimulation duration. **c**, Left: representative example of tail angle traces. Right: maximum absolute tail angle versus median TBF of all locomotor episodes elicited by electrical stimulation classified as forward swims (blue) or struggles (magenta) (13 fish, 164 stimulations, 786 episodes). Spontaneous episodes are shown as black dots. **d**, Distribution of forward index for all simulations applied. **e1**, Calibration experiments where the fluorescence signal was used to evaluate the spread of the electrical field. Scale bars, 10 µm. **e2**, Scheme of the effective spread of the electrical field (5 fish, 3 electrodes, 10 stimulations). **f**, Location of all stimulation sites investigated color-coded using the median forward index. The dashed line represents the MLR location covering all stimulation sites with a median forward index above 0. **g**, Location of the MLR in larval zebrafish in reference to *Tg(vglut2:DsRed)* larvae of the mapzebrain atlas. Scale bars, 50 µm. Maximum projection Z-stacks are schematized in the bottom right corner with squares including multiple lines; single optical sections are schematized in the bottom right corner with squares including a single line. **h**, Z-stack projection of the MLR region in *Tg(vglut2:DsRed)* (**h1**) and *Tg(gad1b:GFP)* (**h2**) transgenic lines. Scale bars, 20 µm. **i1**–**i4**, Distribution of retrogradely labeled MLR neurons (**i1**, red) in the vicinity of neurons immunoreactive to DBH (white) (**i2**) and ChAT (yellow) (**i3**,**i4**). This experiment was successfully replicated twice. **j1**, Midbrain and hindbrain region of 6-dpf *Tg(UAS:kaede)* larvae coinjected with the sgRNA targeting a cut site upstream of the *drd1a* locus. **j2**, *Tg(UAS:mScarlet)* expression pattern under the control of the *Tg(GAL4FF)*^*uot17*^ driver. Scale bars, 40 µm. **k**, Location of ROIs inside the MLR from *n* = 31 fish used for light sheet functional imaging. **l**, Violin plot showing the proportions of MLR neurons whose activity was correlated with motor activity (left) and MLR neurons whose activity was correlated with vigor of the bout (right) (the line is the median value). **m**, Example traces from vigor-correlated MLR neurons of larva exposed to OMR stimulation. **n**, Top: calcium activity of a representative vigor-correlated neuron in one fish plotted against the vigor regressor of the locomotor output for spontaneous (left) and visually evoked (right) swim bouts. Bottom: regression analysis for all 1,235 vigor-correlated MLR neurons for all 31 fish for spontaneous (left) and visually evoked (right) swim bouts. Each black line represents the correlation per fish of all vigor-correlated neurons in the MLR locus. The blue line represents the correlation of all 1,235 vigor-correlated MLR neurons across the 31 fish. A, anterior; P, posterior; D, dorsal; V, ventral; L, left; R, right; M, medial; Lat, lateral. Source data

We monitored the behavioral responses of 6-day post-fertilization (dpf) head-embedded, tail-free larvae exposed to 2–40-s stimulation trains of 2-ms pulse duration occurring at 5–20 Hz frequency and 0.1–2 µA intensity (Fig. 2a). Fish responded either with coordinated symmetric forward swims (Fig. 2b, blue trace), uncoordinated asymmetric tail bends resembling escape or struggle (Fig. 2b, magenta trace) or a mix of both occurring sequentially as distinct motor episodes within the same bout (Fig. 2c, left). We manually segmented and classified each episode as either forward swim or escape or struggle (Methods). The maximal tail bend amplitude (TBA) and the median tail beat frequency (TBF) best differentiated forward swimming from escape or struggle episodes (Fig. 2c, right). For each stimulation, we defined a forward index as the difference of occurrence of forward versus escape or struggle episodes (forward index = (number of forward swim episodes − number of escape or struggles) / number of all episodes; Fig. 2d). Using the same stimulation protocol in 6-dpf transgenic *Tg(elavl3:GCaMP6f)*^30^ larvae expressing GCaMP6f in all neurons, we determined that each electrical pulse can stimulate neurons distributed over a sphere of approximately 28 µm in diameter on average (Fig. 2e). For each position of the microelectrode sampled, we calculated a median forward index over all stimulations and then mapped each three-dimensional (3D) position to a reference brain space based on the mapzebrain atlas^31^ (Fig. 2f). We defined the MLR as the brain stem region consisting of all stimulation sites with a positive median forward index. Consequently, the MLR locus corresponded to a confined area of approximately 40 µm in diameter located at the boundary between the isthmus and rhombomere 1 (r1), which was medial and dorsal to the locus coeruleus (LC) (Fig. 2f,g).

**Extended Data Fig. 1:**
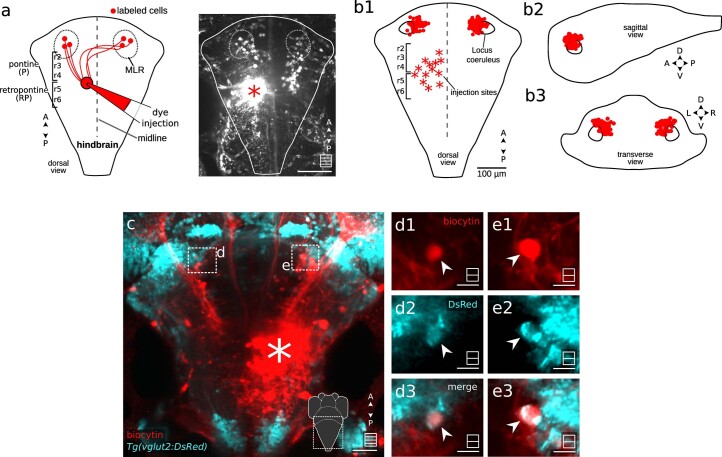
Backfilling of MLR neurons using injections of biocytin in the reticular formation. **a**. Left panel: schematic representation of the retrograde tracing experimental design in which biocytin was unilaterally injected in the pontine and retropontine reticular formation between rhombomere 2 and 6 (r2-r6) to reveal neurons in the MLR locus projecting in the reticular formation (r, rhombomere). Right panel: a typical Z projection stack acquired 2 hours after the backfill injection (asterisk) shows neurons with a soma located in the MLR area (dorsal view, white dash line circle, radius of ~ 40 µm centred on the MLR). Scale bar is 100 µm. **b**. Location of all retrogradely labeled neurons found in the MLR locus denoted in panel a mapped onto a common brain space (11 fish, 156 cells, neurons backfilled contralateral to injection site: 8.00 ± 4.6, ipsilateral: 6.18 ± 4.6) in the dorsal (b1), sagittal (b2) and transverse (b3) planes. The number of cells found ipsi- and contra-laterally to the injection site are not statistically different (one-sided t-test; t(19.8) = 0.9 p = 0.35). **c**, Some of the retrogradely labeled neurons were glutamatergic as shown by overlap with the Tg(vglut2:DsRed) transgenic larvae: a maximal Z-stack projection over 10 µm revealed retrogradely-labeled neurons (red) shown in the dorsal plane together with DsRed+ cells (cyan). Biocytin injection is denoted with an asterisk. Scale bar is 25 µm. **d**, **e**, Detailed view of regions denoted in panel e and corresponding to single planes acquired at different depths. Arrowheads indicate putative glutamatergic retrogradely-labeled neurons. This experiment was successfully replicated 7 times. Scale bar is 5 µm.

The MLR includes glutamatergic, cholinergic and GABAergic neurons^9^. Using the transgenic lines *Tg(vglut2:DsRed)* and *Tg(gad1b:GFP)*, we confirmed that glutamatergic (Fig. 2h1) and GABAergic (Fig. 2h2) neurons belong to the rostral lateral and caudal medial portion of the MLR locus, respectively. Retrograde tracing experiments using unilateral injections of biocytin between rhombomeres 2 (r2) and 6 (r6) confirmed that MLR neurons projected equally ipsilaterally and contralaterally relative to the injection site (Extended Data Fig. 1a,b). Using this protocol in the *Tg(vglut2:DsRed)* larvae revealed that numerous MLR neurons were glutamatergic (Extended Data Fig. 1c–e). A cluster of MLR neurons (Fig. 2i1), backfilled from the injection of tracer in the P reticular formation, was medial to dopamine β-hydroxylase (DBH)-immunoreactive cells of the LC (Fig. 2i2) and near a cluster of ChAT immunoreactive cells (Fig. 2i3,i4), presumably belonging to the laterodorsal tegmental nucleus (LDT) (arrow)^32^. In mammals, salamanders and lampreys, dopaminergic neurons from the substantia nigra pars compacta directly activate MLR neurons through the dopamine receptor D_1_ (ref. ^33^). Thus, we hypothesized that a subset of MLR neurons should express the dopamine receptor D_1_. We used three sets of guide RNAs (gRNAs) to perform CRISPR–Cas9-mediated genome editing targeting the genetic locus of the dopamine receptor D_1a_ (*drd1a*) to insert a GAL4 (ref. ^34^) cassette and label *drd1a*-expressing cells. Both transient (Fig. 2j1) and stable (Fig. 2j2) expression of a reporter after targeting the *drd1a* locus revealed numerous neurons expressing the D_1_ receptor in the MLR locus.

To test whether the MLR locus includes neurons whose activity is motor-correlated, we then examined the activity of neurons in this locus in thirty-one 6–8-dpf transgenic *Tg(elavl3:GCaMP6s)* larval zebrafish performing optomotor response (OMR)^35^. The MLR locus contained 110 ± 20 neurons (31 fish; values given as mean ± s.d. from hereafter): the activity of 30.8 ± 16,8% of these cells (~ 33 cells per fish) was motor-correlated. Out of these, two-thirds (64.2 ± 23.8%) scaled with the vigor of the swim bout (about 22 neurons; Fig. 2k–m, 31 fish). Interestingly, vigor-correlated neurons in the MLR locus were active during both spontaneous bouts and for visually evoked bouts (Fig. 2n), suggesting that MLR neurons are active to trigger locomotion independently of the sensory context.

Altogether, our approach uncovered the locus of the MLR in larval zebrafish as a PP area dorsomedial to the LC that contains glutamatergic, GABAergic and cholinergic neurons. Stimulation of neurons in this area is effective at triggering forward locomotion. Population calcium imaging provides evidence for motor-correlated neurons in the MLR locus and reveals that a large fraction of MLR neurons are active during spontaneous and visually evoked locomotion as a function of the vigor of the bout.

### MLR sets the kinematics of locomotion

**Fig. 3:**
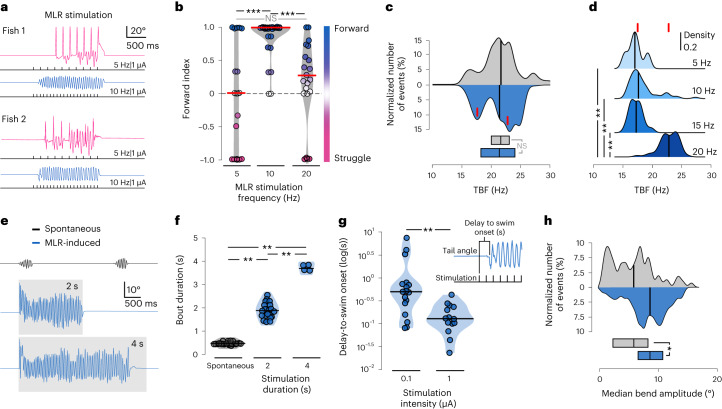
MLR stimulation parameters set the duration, time onset, amplitude and locomotor frequency of forward swimming. **a**, Representative behavioral responses to MLR stimulation at different frequencies in two different larvae. **b**, Forward index for all the stimulation sites according to stimulation frequency (10 fish and 64 stimulation trials; 5 Hz: *n* = 19 stimulations, −0.06 ± 0.8; 10 Hz: *n* = 26 stimulations, 0.919 ± 0.24; 20 Hz: *n* = 19 stimulations, 0.27 ± 0.55 s; Kruskal–Wallis test, *χ*^2^ = 105.85, d.f. = 3, *P* < 0.001, two-sided Wilcoxon pairwise comparisons: 5 Hz versus 10 Hz: ***P < 0.001; 5 Hz versus 20 Hz: *P* = 0.17; 10 Hz versus 20 Hz: ***P < 0.001). *P* values were adjusted using the Bonferroni method. **c**, Distribution of median TBF on MLR stimulation (the red line represents the peaks of the distributions: 17.5 Hz and 23.2 Hz) compared to spontaneous locomotion (11 fish; spontaneous: 57 episodes, 21.6 ± 3.1 Hz; MLR stimulation: 242 episodes, 21.3 ± 2.9 Hz; Mann–Whitney *U*-test, *W* = 6,721, *P* = 0.7; the boxes at the bottom show the median and 25–75th quantiles). **d**, A 20-Hz MLR stimulation elicited swimming with higher TBF compared to lower stimulation frequency or spontaneous swimming (11 fish; 5 Hz: ten episodes, 17.54 ± 1.31 Hz; 10 Hz: 60 episodes, 18.7 ± 2.34 Hz; 15 Hz: 18 episodes, 17.66 ± 1.07 Hz; 20 Hz: 154 episodes, 23.1 ± 1.618 Hz; Kruskal–Wallis test, *χ*^2^ = 6237, d.f. = 31, *P* < 0.001, two-sided Wilcoxon pairwise comparisons: 5 Hz versus 10 Hz: *P* = 0.3; 5 Hz versus 15 Hz: *P* = 0.9; 5 Hz versus 20 Hz: *P* < 0.001; 10 Hz versus 15 Hz: *P* = 0.2; 10 Hz versus 20 Hz: *P* < 0.001; 15 Hz versus 20 Hz: *P* < 0.001). *P* values were adjusted using the Bonferroni method. The red lines depict the peak values for each of the distributions from **c**. **e**, Example tail angle traces for spontaneous forward swims (black, top trace) and forward swims induced by stimulating the MLR for 2 s (blue, middle trace) or 4 s (blue, bottom trace). The gray box indicates the duration of the train. **f**, Quantification of the duration of forward swims according to the duration of the MLR stimulation (10 Hz, 1 µA), displayed as a violin plot with the black line indicating the median value (8 fish; spontaneous: 27 episodes, 0.41 ± 0.15 s; 2-s stimulation: 19 episodes, 1.91 ± 0.44 s; 4-s stimulation: 4 episodes, 3.65 ± 0.315 s; Kruskal–Wallis test, *χ*^2^ = 38.6, d.f. = 2, **P < 0.001, Wilcoxon pairwise comparisons: spontaneous versus 2-s train stimulation, *P* < 0.001; spontaneous versus 4-s train stimulation, **P < 0.001; 2 s versus 4 s, **P < 0.001). **g**, Violin plot displaying the distribution of the delay to swim onset (the black line represents the median value) (9 fish; all MLR stimulation set at 10 Hz for 2 s; 0.1 µA: 17 episodes, 1.22 ± 1.97 s; 1 µA: 14 episodes, 0.15 ± 0.11 s; Mann–Whitney *U*-test, *W* = 199, *P < 0.001). **h**, MLR-evoked forward swims reliably exhibited larger TBA than spontaneous forward swims (12 fish; spontaneous: 67 episodes, 5.9 ± 3.16°; MLR stimulation: 172 episodes, 8.5 ± 3.8°; Mann–Whitney *U*-test, *W* = 8102, *P < 0.001). In all panels, the black line indicates the median value. Source data

The intensity of MLR stimulation controls the duration and power of locomotion^10^. Therefore, we investigated whether the intensity, train duration and pulse frequency defining MLR stimulation modulated the duration and kinematic parameters of forward swims (Fig. 3). We noticed that stimulating the MLR at 10 Hz was the most effective at inducing forward swimming, while 5 Hz or 20 Hz stimulation often resulted in escape or struggle-like behavior (Fig. 3a,b). This observation is consistent with previous results in lampreys and salamanders showing an optimal frequency of stimulation between 2 Hz and 10 Hz^36,37^. Spontaneous and MLR-induced locomotion exhibited similar median TBF (Fig. 3c). On closer inspection, we noticed that the TBF probability density distribution on MLR stimulation was bimodal (Fig. 3c). We found indeed that MLR-induced forward swims exhibited a median TBF around 17 Hz when the stimulation occurred at frequencies below 15 Hz (Fig. 3d), while 20 Hz MLR stimulation elicited forward swims at 23 Hz (Fig. 3d). Stimulating the MLR for 2 s or 4 s elicited sustained episodes lasting the stimulation train (Fig. 3e,f and Supplementary Video 1). In comparison, spontaneous forward swims recorded between stimulation trials only lasted for a few hundreds of milliseconds (Fig. 3e,f). Furthermore, as previously observed in lampreys and salamanders^36,37^, the delay to locomotor onset was inversely proportional to the stimulation intensity (Fig. 3g). We noticed that MLR-induced forward swims exhibited larger median TBA than spontaneous swims (Fig. 3h). Altogether, our observations indicate that the properties of MLR stimulation impact the duration, time onset, amplitude and locomotor frequency of forward swims.

### MLR recruits ipsilateral and contralateral RSNs but not nMLF neurons

**Fig. 4:**
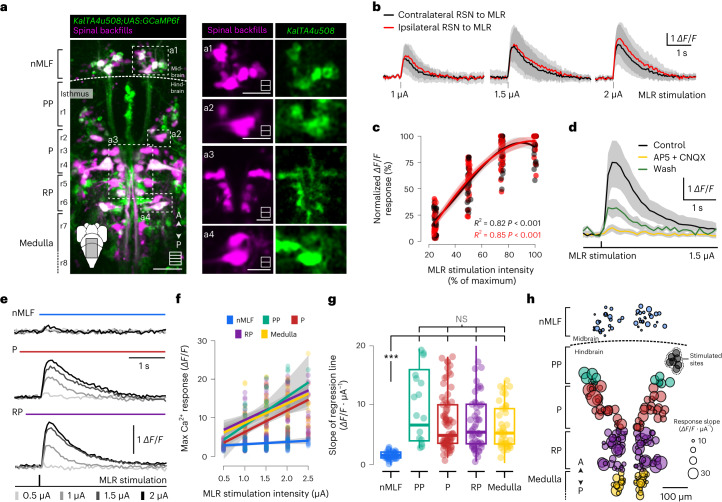
Unilateral MLR stimulation recruits ipsilateral and contralateral hindbrain RSNs but not the nMLF. **a**, Z-stack projection of ten optical sections acquired from 6-dpf *Tg(KalTA4u508;UAS:GCaMP6f)* larvae (green) with RSNs backfilled (magenta). Right: magnified images of the areas denoted in the left. Scales bars, 100 µm (left) and 50 µm (right). **b**, Average calcium response traces of hindbrain RSNs for one example fish in response to increasing MLR stimulation intensities (mean ± s.d., 18 neurons, grouped according to location of the electrode; red, ipsilateral; black, contralateral). **c**, Maximum calcium responses of RSNs pooled according to location either ipsilateral (red) or contralateral (black) relative to the stimulation electrode (5 fish, 53 neurons). Data followed a cubic polynomial function (ipsilateral: *R*^2^ = 0.85, *F*_(112, 13.02)_ = 211.3, *P* < 0.001; contralateral: *R*^2^ = 0.82, *F*_(100, 13.45)_ = 151.1, *P* < 0.001). **d**, Bath application of the glutamatergic antagonists CNQX (70 µM) and AP5 (70 µM) blocked the MLR-induced calcium responses of RSNs (2 fish, 72 cells, mean ± s.d.: 40-min drug incubation, 7.6 ± 2.6% of pre-drug application, after 180 min of washout 23 ± 8% of pre-drug application). **e**, Typical calcium transients of individual neurons in response to increasing intensity of MLR stimulations. **f**, Relationship between maximum calcium response amplitudes and MLR stimulation intensity for an example larva. Each dot represents a neuron. The linear correlation (line) was calculated for all the neurons in each defined region. **g**, Quantification of the slope reflecting the linear correlation between change of fluorescence and MLR stimulation intensity for all neurons tested. The box plots are depicted as the mean (center), first and third quartiles (lower and upper box limits), and minima and maxima (bottom and top whiskers) (9 fish, nMLF: 43 neurons, 0.74 ± 1.4 *ΔF/F* *·* µA^−1^; PP: 12 neurons, 7.8 ± 5.9 *ΔF/F* *·* µA^−1^; P: 72 neurons, 5.1 ± 4.7 *ΔF/F* *·* µA^−1^; RP: 52 neurons, 5.2 ± 5.7 *ΔF/F* *·* µA^−1^; medulla: 38 neurons, 4.2 ± 3.8 *ΔF/F* *·* µA^−1^; Kruskal–Wallis test, *χ*^2^ = 48.2, *P* < 0.001, Wilcoxon pairwise comparisons. *P* values were adjusted using the Bonferroni method: nMLF versus PP: *P* < 0.001; nMLF versus P: *P* < 0.001; nMLF versus RP: *P* < 0.001; nMLF versus medulla: *P* < 0.001; PP versus P: *P* = 0.26; PP versus RP: *P* = 0.26; PP versus medulla: *P* = 0.58; P versus RP: *P* = 0.91; P versus medulla: *P* = 0.58; RP versus medulla: *P* = 0.73). Three asterisks correspond to P < 0.001. **h**, Spatial distribution of RSN size coded using the slope plotted in **g**. MLR stimulation loci are displayed as gray dots. Source data

MLR neurons provide glutamatergic monosynaptic inputs to RSNs in the hindbrain^38^. Unilateral stimulation of the MLR recruits ipsilateral and contralateral RSNs^13,39^. On single-pulse MLR stimulation, the spiking frequency and duration of the RSN response scaled with stimulation intensity^38^. To investigate the functional connectivity between the MLR and RSNs in larval zebrafish, we used the *Tg(KalTA4u508; UAS:GCaMP6f)* transgenic line^40^ in which GCaMP6f is expressed in the spinal-projecting neurons of canonical zebrafish (Fig. 4a). We took advantage of the fact that this transgenic line also labels neurons of the nMLF in the diencephalon (Fig. 4a) to test whether these neurons were recruited with our MLR stimulation protocol. We recorded calcium responses on unilateral single-pulse MLR stimulation (0.5–2 µA amplitude, 2-ms-long) in 6-dpf paralyzed larvae. Such unilateral single-pulse MLR stimulations consistently led to bilateral recruitment of RSNs throughout all stimulation intensities (Fig. 4b,c). These calcium responses were abolished on bath application of the glutamatergic antagonists AP5 (70 µM) and CNQX (70 µM) and partially recovered after washout, confirming the crucial role of glutamatergic inputs from the MLR onto RSNs (Fig. 4d). Furthermore, the amplitude of the RSN calcium response scaled with the intensity of MLR stimulation (Fig. 4e–h). In contrast, there was no obvious activation of diencephalic spinal-projecting neurons of the nMLF, despite being in proximity to the stimulation electrode (Fig. 4e–h). Altogether, our results confirm that the MLR in larval zebrafish provides bilateral glutamatergic inputs to hindbrain RSN and drives forward locomotion without recruiting nMLF neurons.

### The MLR differentially recruits distributed hindbrain V2a RSNs

**Fig. 5:**
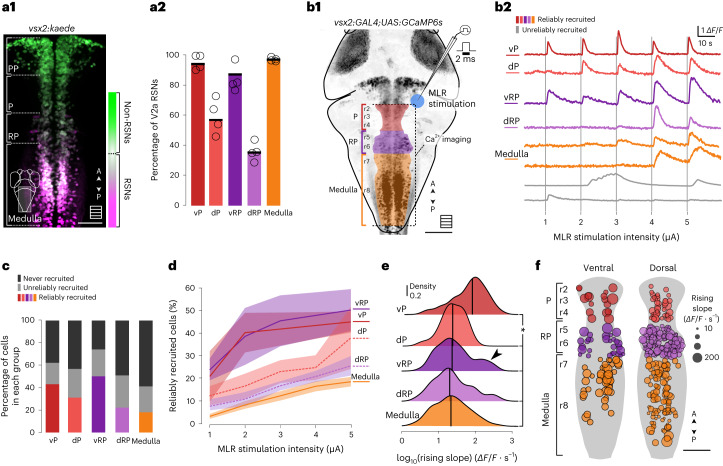
Single-pulse MLR stimulations gradually recruit a subset of V2a RSNs. **a**, Distribution and density of RSNs in the V2a neuronal population. **a1**, Example image of the hindbrain of 5-dpf *Tg(vsx2:Kaede)* larval zebrafish after photoconverting Kaede protein in the rostral spinal cord. Scale bar, 50 µm. **a2**, Mean proportion of V2a RSNs (4 fish). **b1**, Schematic illustration of the experiments investigating V2a neuron recruitment in response to MLR stimulation in paralyzed *Tg(vsx2:GAL4;UAS:GCaMP6s)* transgenic 6-dpf larval zebrafish. **b2**, Typical calcium traces of reliably recruited V2a neurons in the different anatomical regions investigated (top, colored traces: vP, dP, vRP, dRP and medulla). The gray traces at the bottom represent neurons not labeled as reliably recruited. **c**, The recruitment of V2a neurons fell into three groups: reliably recruited (the color corresponds to reliably recruited neurons in a given anatomical area); unreliably recruited (gray); and not recruited (black) (5 fish, total 1,781 neurons; vP: 42 neurons; dP: 96 neurons; vRP: 88 neurons; dRP: 475 neurons; medulla: 1,080 neurons). **d**, Proportions of reliably recruited V2a neurons (mean ± s.d.) responding at each MLR stimulation intensity (proportions are relative to the numbers of V2a neurons in each anatomical group defined in **b**). **e**, Distribution of calcium transient rising slopes in reliably recruited neurons. The line is the median value (median ± s.d., 5 fish; vP: 18 neurons, 76 events, 64.4 ± 67.6 *ΔF/F* *·* s^−1^; dP: 29 neurons, 87 events, 23.4 ± 20.2 *ΔF/F* · s^−1^; vRP: 44 neurons, 177 events, 30.6 ± 43.3 ΔF/F · s^−1^; dRP: 100 neurons, 326 events, 27.1 ± 30.7 *ΔF/F* · s^−1^; medulla: 183 neurons, 583 events, 24 ± 27.3 *ΔF/F* · s^−1^; two-sided Kruskal–Wallis test, *χ*^2^ = 141.68, *P* < 0.001. Wilcoxon pairwise comparisons: vP versus dP: *P* < 0.05; vP versus vRP: *P* < 0.01; vP versus dRP: *P* < 0.01; vP versus medulla: *P* < 0.001; dP versus vRP: *P* = 1.0; dP versus dRP: *P* = 1.0; dP versus medulla: *P* = 1.0; vRP versus dRP: *P* = 1.0; vRP versus medulla: *P* = 1.0; dRP versus medulla: *P* = 1.0). *P* values were adjusted using the Bonferroni method. **f**, Location of reliably recruited V2a neurons (dot size scaled with the median of all computed rising slopes for each neuron). Source data

Because the transgenic line *Tg(KalTA4u508)*^*u508Tg*^ drives expression solely in spinal-projecting neurons whose axon projects in the medial fasciculus, it does not allow for investigation of medullary V2a RSNs that project their descending axon toward the lateral fasciculus^41^. Therefore, we next sought to investigate whether the MLR also recruits V2a RSNs (Fig. 5). Because not all hindbrain V2a neurons are RSNs^17,18,41,42^, we first quantified the proportions of V2a RSNs in each subdivision of the hindbrain (nomenclature of Fig. 1) by photoconverting Kaede protein-expressing axons in the rostral spinal cord of 4.5-dpf *Tg(vsx2:Kaede)*^43^ transgenic larvae. To identify RSNs in the V2a population, we illuminated the rostral spinal cord with ultraviolet light to target the descending RSN axons and waited for the photoconverted protein to diffuse to their somata in the hindbrain (Fig. 5a1). We found only 57% of all V2a neurons in the dorsal P (dP) to be RSNs (57.5 ± 18.1%) and 35% in the dorsal RP (dRP) region (35.5 ± 6.1%) (Fig. 5a2). In contrast, most V2a neurons were RSNs in the ventral P (vP) nucleus (95.6 ± 5.1%, four fish), ventral RP (vRP) (84.1 ± 9.5%) and medulla (96.6 ± 1.8%). Overall, the medulla contained most (60%) V2a RSNs.

**Extended Data Fig. 2:**
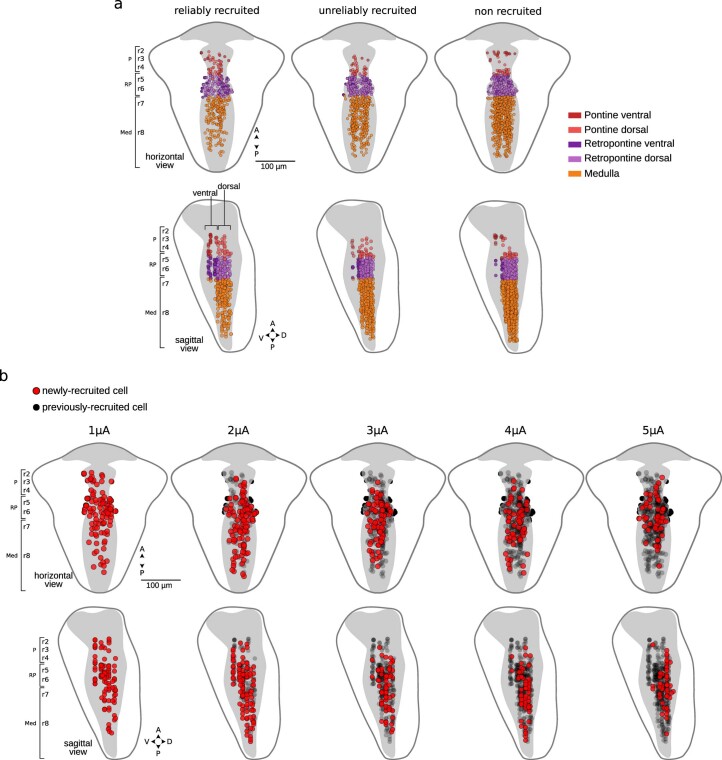
MLR recruitment of V2a neurons. **a**, Location of V2a neurons reliably recruited (n = 391), unreliably recruited, (n = 553) and non-recruited (n = 837) throughout the pontine, retropontine and medulla in 5 fish. Horizontal (top) and sagittal (bottom) views are shown. **b**, Hindbrain location of the reliably recruited V2a neurons activated by MLR stimulation at increasing intensities (1 to 5 µA) (391 neurons, 5 fish). The red dots represent newly recruited neurons at a particular stimulation intensity. Gray dots show neurons previously recruited at lower intensity (newly-recruited neurons). Source data

To test whether the MLR drives ipsilateral and contralateral V2a RSNs in larval zebrafish, we investigated their response in *Tg(vsx2:GAL4;UAS:GCaMP6s)*^18^ transgenic larvae on unilateral and single-pulsed MLR stimulations (1–5 µA amplitude, 2-ms pulse; Fig. 5b1,b2). We found that such unilateral stimulation of the MLR evoked excitatory responses in V2a neurons throughout the hindbrain on both sides from the P region to the medulla (Extended Data Fig. 2a). On stimulation of graded intensities (Fig. 5b2), a subset of hindbrain V2a neurons reliably responded above a given threshold (reliably recruited neurons depicted in color were on average 22% of all V2a RSNs recorded; Extended Data Fig. 2b), others either did not consistently respond (unreliably recruited depicted in gray were on average 31% of all V2a RSNs recorded) or never responded (non-recruited not shown were on average 47% of all V2a RSNs recorded). We focused our analysis on reliably recruited cells because they probably receive excitatory inputs from the MLR and contribute to the motor output (*n* = 391 of 1,781 recorded neurons). Half of V2a RSNs were reliably recruited in the ventral portion of the P and RP areas (Fig. 5c; vP: 42.8%; dP: 32.0%; vRP: 50.0%; dVP: 22.7%) and their recruitment occurred at the lowest stimulation intensity tested (Fig. 5e). We investigated whether the calcium responses of reliably recruited V2a RSNs exhibited different rising slopes on MLR stimulation. Responses of V2a RSNs in the vP region and RP areas (Fig. 5e, subset marked with the arrowhead) showed consistently large rising slopes, larger than the ones observed in any of the other regions (Fig. 5f), suggesting an effective and prompt coupling between MLR and ventrally located V2a RSNs. In contrast to RSNs in the vP and RP areas, only a subset of V2a RSNs were reliably recruited in the medulla (Fig. 5c, 17%). The recruitment of these medullary V2a RSNs occurred in a graded manner as a function of MLR stimulation intensity (Fig. 5d–f), suggesting a weaker coupling from MLR neurons onto medullary RSNs than vP and RP RSNs.

### MLR neurons differentially project to hindbrain RSNs

**Fig. 6:**
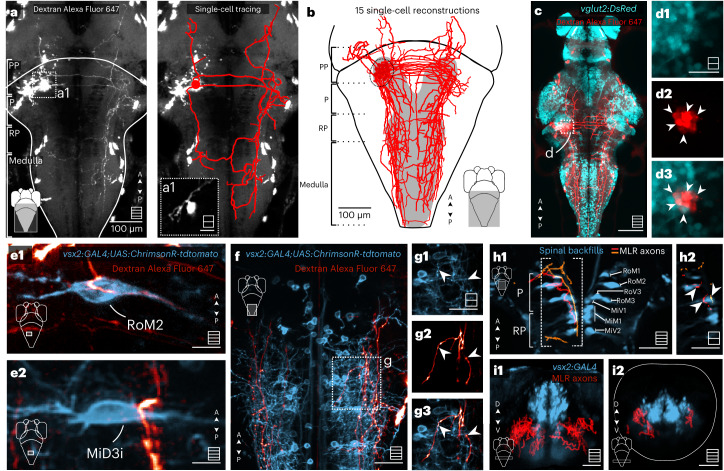
MLR neurons project bilaterally to V2a RSNs forming putative axosomatic synapses in the P and RP areas and axodendritic synapses in the medulla. **a**,**a1**, Left: forsal view of a Z-stack showing the projection pattern of an MLR neuron that was electroporated with Dextran Alexa Fluor 647. Right: reconstruction of the arborization pattern. **b**, Reconstruction of electroporated neurons with somata located in the MLR locus (7 fish, 15 neurons). **c**, The MLR contains many glutamatergic neurons projecting to the reticular formation. Scale bar, 100 µm. **d1**–**d3**, The single plane revealed that many MLR electroporated neurons (red) were glutamatergic, as shown by the overlap with the *Tg(vglut2:DsRed)* transgenic line (cyan) (7 fish, 14 neurons). Scale bar, 20 µm. **e**, Electroporation of MLR neurons (red) in *Tg(vsx2:GAL4;UAS:ChrimsonR-tdtomato)* (blue) transgenic fish. Putative connections of the MLR to an ipsilateral P RSN (**e1**) and a contralateral RP RSN (**e2**). Scale bars, 10 µm. **f**,**g**, Z-stack of the medulla region (**f**) showing the typical projection pattern on the MLR neurons to the lateral dendritic area (**g1**–**g3**). **h1**, Dorsal view of a Z-stack of spinal backfills labeling (blue) and two examples of MLR neurons (orange and red) taken from the single-cell atlas mapzebrain. **h2**, Single plane of the region denoted in **h1** showing the axons of the MLR neurons reaching the soma of the P and RP RSNs. **i**, Coronal view of a Z-stack of V2a neurons (blue) and all MLR neurons recovered from the single-cell atlas mapzebrain (red) in the RP region (**i1**) and caudal medulla (**i2**). Scale bars, 40 µm. In all panels, maximum projection Z-stacks are schematized in the bottom right corner, with squares including multiple lines and single optical sections schematized with squares including a single line. Source data

To identify the possible neural substrate underlying the differential recruitment pattern of V2a RSNs by the MLR, we labeled MLR neurons by performing focal electroporations^44^ within the MLR locus. We traced the skeleton of each electroporated cell and registered it to a common reference brain space^31^ (Fig. 6a). This approach revealed that MLR neurons send ipsilateral and contralateral axons descending ventrally toward the medulla through the lateral longitudinal fasciculus (Fig. 6a,b). The commissural axon passed through the contralateral MLR before descending caudally to the reticular formation (Fig. 6a,b). The axon of many MLR neurons projected down to the caudal-most medulla (Fig. 6b). Focal electroporation in the MLR locus in *Tg(vglut2:DsRed)* larvae confirmed that MLR glutamatergic neurons projected equally ipsilaterally and contralaterally relative to the injection site (Fig. 6c,d). The observation of direct projections from glutamatergic MLR neurons onto the reticular formation is consistent with the results of our pharmacological experiment (Fig. 4).

To decipher which cellular compartments of V2a RSNs were targeted by MLR neurons, we performed focal MLR neuron electroporation in transgenic *Tg(vsx2:GAL4;UAS:ChrimsonR-tdtomato)* larvae. We noted interesting differences in the innervation received by P and RP versus medullary V2a RSNs (Fig. 6e–g). While MLR neurons made axosomatic contacts onto ventrally located V2a RSNs in the P and RP regions (Fig. 6e and Supplementary Video 2), they did not form any axosomatic contacts but formed solely axodendritic connections onto medullary V2a RSNs (Fig. 6f,g).

**Extended Data Fig. 3:**
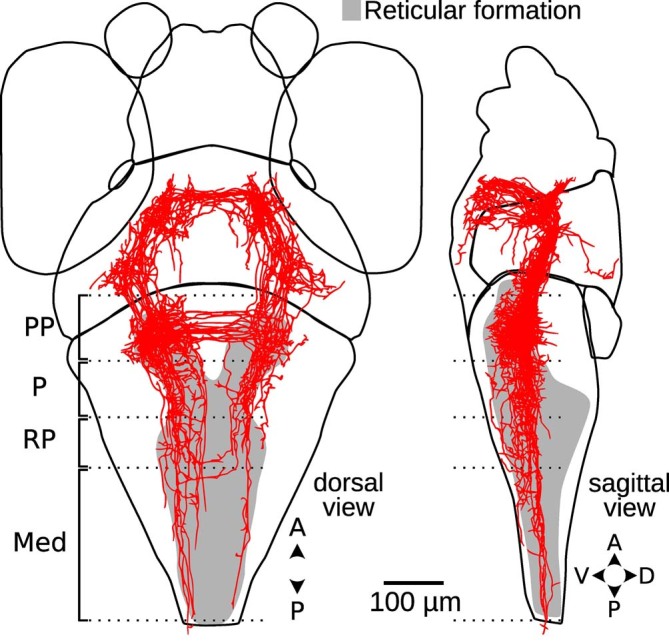
MLR neurons recovered from single cell zebrafish brain atlas. 3D rendering of neurons with soma located in the MLR from the mapzebrain single-neuron atlas (n = 23 neurons).

To confirm the morphology of MLR neurons using another approach, we used a virtual single-cell tracing by interrogating the cellular resolution brain atlas of mapzebrain. As observed in our electroporation experiments, we found that neurons whose soma was in the MLR locus systematically projected on the ipsilateral and contralateral reticular formation (*n* = 23 of 23), sometimes reaching down to the most caudal portion of the medulla (Extended Data Fig. 3). A subset of MLR neurons sent their axonal projection ventrally and medially to form putative axosomatic connections with ventral RSNs in the P and RP regions (5 of 23 neurons; Fig. 6h1,h2). In contrast, as we observed in our focal electroporation experiments, in the medulla, the axons of MLR neurons run ventrally and laterally throughout the reticular formation, not reaching the medial stripe where V2a somata are located (20 of 23 neurons; Fig. 6i1,i2 and Extended Data Fig. 3). These analyses based on stochastic single-cell labeling in the brain atlas further support a differential targeting of MLR neurons onto specific cellular compartments of V2a RSNs in the P and RP regions versus the medulla, which can well explain the differential coupling observed between MLR neurons and P and RP (strong) versus medullary (weak) V2a RSNs (Fig. 5).

### Distinct maps of V2a medullary neurons for forward locomotion and steering

**Fig. 7:**
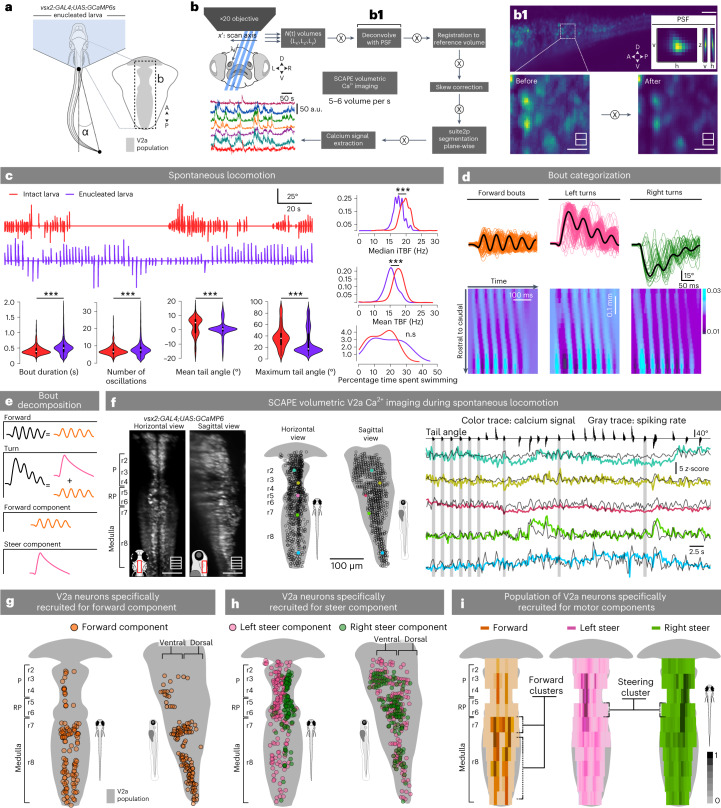
Whole-population brain stem V2a neurons recruitment during spontaneous swimming using high-speed, single-objective light sheet microscopy. **a**, Schematic illustration of the experiment investigating hindbrain V2a neurons recruitment during spontaneous locomotion in enucleated 6-dpf *Tg(vsx2:GAL4;UAS:GCaMP6s)* larvae. **b**, Pipeline workflow developed for the SCAPE microscopy adapted from refs. ^45^ and ^46^. **b1**, Example of deconvolved image using the system’s experimental PSF (Methods). **c**, Top left: typical tail angle trace exhibited by intact (red, top) and enucleated (violet, bottom) larvae during recordings (intact: 5 larvae, 512 swim bouts; enucleated: 12 larvae, 3,946 swim bouts). Bottom left: violin plots depicting the distribution of bout duration (intact: 432 ± 340 ms; enucleated: 532 ± 340 ms; enucleated > intact with *P* < 0.001, Wilcoxon rank-sum test), number of oscillations (intact: 7.51 ± 6.3; enucleated: 8.3 ± 6.3; enucleated > intact with *P* < 0.001, Wilcoxon rank-sum test), mean tail angle (intact: 2.14 ± 6.6°; enucleated: 0.45 ± 6.6°; enucleated = intact with *P* < 0.001, Wilcoxon rank-sum test) and maximum absolute tail angle (intact: 36.43 ± 23.48°; enucleated: 27.15 ± 23.48°; enucleated < intact with *P* < 0.001, two-sided Wilcoxon rank-sum test) of each episode, in each condition. The box plots depict the mean (center), first and third quartiles (lower and upper box limits), and minima and maxima (bottom and top whiskers). Right: density distribution of TBF across the two conditions computed as either the median iTBF or mean TBF. Mean iTBF was greater in intact larvae (intact: 17.12 ± 2.35 Hz; enucleated: 15.11 ± 2.35 Hz; *P* < 0.001, Wilcoxon rank-sum test), as well as median iTBF (intact: 19.68 ± 1.75 Hz; enucleated: 17.60 ± 1.75 Hz; *P* < 0.001, Wilcoxon rank-sum test; three asterisks correspond to *P* < 0.001). **d**, Top: tail angle traces of swimming episodes classified as forward (orange), left turns (magenta) and right turns (green) in one example experiment (1 fish, one recording, *n* = 311 bouts classified as 117 forward, 127 left turns, and 29 right turns; note that 38 bouts did not fall in any category and were discarded from further analysis). Bottom: tail curvature plots for example bouts of the three types. The curvature value of each segment (from rostral to caudal, in *y*) is represented in the color scale as a function of time (in *x*). **e**, Schematics of bout type decomposition into kinetic components. We assumed that a turn was composed of a directionality bend (steer component) executed in tandem with symmetric oscillating bends (forward component). **f**, Left: example volume image (as the average of multiple time steps) of a 6-dpf larval zebrafish after pipeline processing. Middle: position of example neurons (colored dots) among the population of all recorded V2a neurons (unfilled dots), in dorsal and sagittal views. Right: *ΔF/F* (colored trace) and inferred spike rate traces (gray) of the example neurons during several swim bouts. The periods during which the larva swam forward are shown as gray rectangles. *ΔF/F* and spike rate traces are represented as their *z*-score. **g**, Distribution of V2a neurons in a representative fish active during both forward bouts, left and right turns, corresponding to the forward component neurons (orange circles, 74 of 844 neurons, 8.9%). **h**, Distribution of V2a neurons for a representative fish active during left (pink, 327 of 844, 38.7%) or right turns (green, 223 out of 844, 26.4%) but not forward swim bouts. **i**, Density distribution of V2a neurons recruited for the forward or steering component (3 fish, median ± s.d.; forward component = 8.9 ± 3.2%; left steering component = 36.5 ± 1.5%; right steering component = 29.5 ± 4.5%). Source data

To understand how MLR neurons recruit V2a RSNs to produce forward locomotion, we first investigated how hindbrain V2a neurons were recruited during spontaneous voluntary locomotion (Fig. 7). To achieve the high-speed volumetric imaging required to do so, we built a swept confocally aligned planar excitation (SCAPE) microscope based on a previous design^45,46^. This microscope enabled us to record calcium activity of the entire V2a hindbrain population at 5.55 volumes per second while recording tail movement of 6-dpf head-fixed, tail-free *Tg(vsx2:GAL4;UAS:GCaMP6s)* transgenic larvae (Fig. 7a,b). We optimized the resolution and cell detection accuracy within the dense V2a population by developing an open-source pipeline to preprocess the large imaging dataset that relies on deconvolution and motion correction of volumetric images (Figure 7b1 and Methods). We enucleated the larvae at 3-dpf to avoid eliciting visual responses due to light sheet scanning. Enucleated larvae displayed spontaneous forward and turn bouts whose properties were similar to the ones of intact larvae (Fig. 7c,d). Forward bouts only consisted of what we refer to as the forward component, defined as a symmetrical undulatory tail movement typically including 3–4 oscillations (Fig. 7d,e, orange trace). In contrast, we decomposed the turn bouts in a steering component that underlies the change of direction (left or right; Fig. 7d,e, magenta trace) and a forward component (Fig. 7d,e, orange trace). We inferred the neuron spiking rate from the calcium signal using a spike deconvolution method^47^ (Fig. 7f, gray traces). We identified neurons recruited for each bout type when their spiking rate was statistically higher during the corresponding bouts than during resting periods.

To investigate which V2a RSNs were active for forward locomotion versus steering, we defined forward-component neurons as cells that were active during both forward and turn bouts (Fig. 7g,i). Forward-component neurons corresponded to a small fraction of the V2a population, located medially along the hindbrain, from r2 to r8, apart from r6 where they were absent (Fig. 7g,i). In the medulla, forward-component neurons formed well-defined medial stripes distributed in two symmetrical clusters, one rostral in r7 and one caudal in r8 (Fig. 7g,i). In contrast, steering-component neurons only recruited for turns (but not forward swims) constituted a larger fraction of the V2a population ipsilaterally distributed relative to the turning side and predominantly located in r2–r6 and in the rostral medulla in r7 (Fig. 7h,i). Interestingly, one dense cluster of steering-component neurons was located in r6, precisely where no V2a neurons were found to be associated with the forward component (Fig. 7i). Our volumetric imaging data show that the medulla houses specific clusters of V2a RSNs active during the forward component of spontaneous locomotion.

### Medullary V2a RSNs encode locomotor duration and frequency

**Fig. 8:**
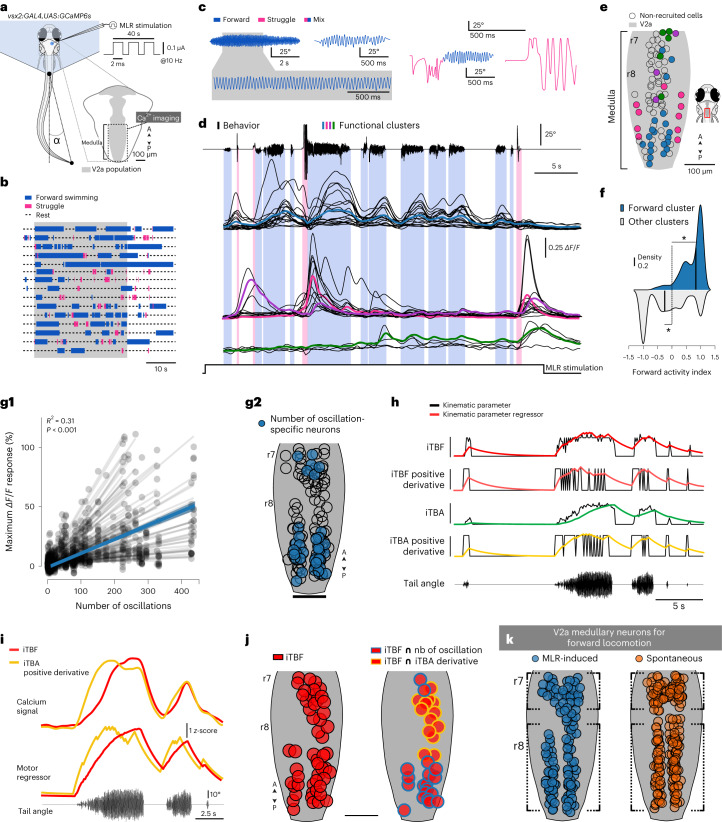
Medullary V2a RSNs are specifically recruited during MLR-induced forward swimming and encode the number of oscillations, TBF and amplitude. **a**, Schematic illustration of the experiment investigating medullary V2a RSN recruitment during MLR-induced forward swimming. **b**, Representation of behavioral periods during all experiments investigated; the color depicts the behavior type (blue: forward episode; pink: escape or struggle). **c**, Example bouts elicited during a 40-s MLR stimulation corresponding to either pure forward swimming (top trace), bout of mixed episodes (bottom left) or pure escape or struggle. **d**, Tail angle trace and calcium activity of the functional clusters of medullary V2a RSNs active during locomotion for an example fish (mean activity in the color trace, individual traces in black). **e**, Location of neurons in the medulla in dorsal view, color-coded according to the functional cluster. The empty circles represent neurons not active. **f**, Medullary V2a RSNs in the forward clusters were recruited more during forward swimming than during struggle behavior. Forward activity index calculated from calcium activity as (forward activity index = average (maximum *ΔF/F* during forward episodes) − average (maximum *ΔF/F* during escape or struggle) / average (maximum *ΔF/F* during forward episodes) + average (maximum *ΔF/F* during escape or struggle)). A positive index indicates an average maximum *ΔF/F* higher during forward than during escape or struggle behavior (5 fish; forward cluster: 1,438 cells (mean ± s.d.): 0.71 ± 0.37, one-sided *t*-test against μ = 0; *t*(1,437) = 72 *P < 0.001; rest of the clusters: 2,603 cells: −0.17 ± 0.72, one sample *t*-test against μ = 0, *t*(6,626) = −12.6, *P < 0.001). **g1**, Linear regression between the maximum *ΔF/F* of individual V2a RSNs from the forward cluster and the number of oscillations of the forward episodes (*n* = 132 neurons in *n* = 5 fish, *n* = 8 trials; the gray lines represent the regression for individual neurons, the blue line the regression for all neurons). **g2**, Location of neurons in the forward cluster; in blue are the ones whose response amplitude correlated the most with the number of oscillations (blue filled circles, *n* = 38 of 132 neurons in *n* = 5 fish, *n* = 6 of 8 trials, *P* < 0.12 and correlation coefficient ≥ 0.8; uncolored circles represents the other neurons). **h**, Motor regressors encoding distinct kinematic parameters: the iTBF, the iTBA and their binary positive derivative. Raw trace (black) and corresponding regressor (color) of each motor feature. Bottom trace: corresponding tail angle. **i**, Top: Traces from two example neurons that were recruited during the forward episodes and whose calcium activity differed during the episode. Middle: two motor regressors that best recapitulated the calcium activity of the two neurons above (matching color codes). Bottom: corresponding tail angle trace. **j**, Left: subsets of V2a medullary RSNs encode the iTBF (*n* = 5 fish, *n* = 6 experiments, *n* = 59 of 89 neurons). Right: distribution of V2a RSNs whose activity encoded TBF and either an increase in TBA (yellow outline) or number of oscillations (blue outline, from **g2**). **k**, Activity map comparison of medullary V2a RSNs obtained during the forward component of spontaneous locomotion (Fig. 7) and MLR-induced forward locomotion (Fig. 8g2). Note the similarity of the forward-component clusters located in the rostral and caudal medulla (dotted brackets). Source data

To investigate whether forward-component medullary V2a RSNs translate the MLR command into graded forward locomotion, we tested whether their activity scales with locomotor frequency or amplitude during MLR-induced forward locomotion (Fig. 8). We performed calcium imaging with optical sectioning in head-fixed tail-free *Tg(vsx2:GAL4;UAS:GCaMP6s)* zebrafish larvae during forward locomotion elicited by prolonged MLR train stimulations (0.1 µA; 10 Hz; 40-s train) (Fig. 8a). Such MLR stimulations elicited unusually long forward episodes lasting up to 20 s and containing hundreds of oscillations (Fig. 8b,c). As the activity of medullary V2a RSNs was diverse, we clustered them based on their calcium traces (Supplementary Video 3). We identified a cluster of medullary V2a RSNs that were specifically recruited during forward swims (Fig. 8d, referred to as forward cluster, blue trace) and another specifically recruited during escape or struggle-like behaviors (Fig. 8d, magenta and violet trace). Medullary V2a RSNs in the forward cluster were medial whereas the ones active during escape or struggle-like behaviors were lateral (Fig. 8e). Medullary V2a RSNs in the forward clusters were recruited more during forward swimming than escape or struggle-like behaviors (Fig. 8f). Interestingly, we noticed that the proportion of V2a medullary neurons of the forward cluster during prolonged MLR train stimulations was similar to the proportion of medullary neurons we previously found reliably recruited by the single-pulsed MLR stimulations (forward cluster: 18.32 ± 7.32%, 26.4 ± 12.6 of 228 ± 35.9 neurons per fish, a total of 1,140 neurons out of 5 fish recorded; Fig. 5: reliably recruited V2a neurons: 22% of medullary V2a neurons), suggesting that the same population of V2a RSNs receiving inputs from MLR neurons elicits forward swimming.

**Extended Data Fig. 4:**
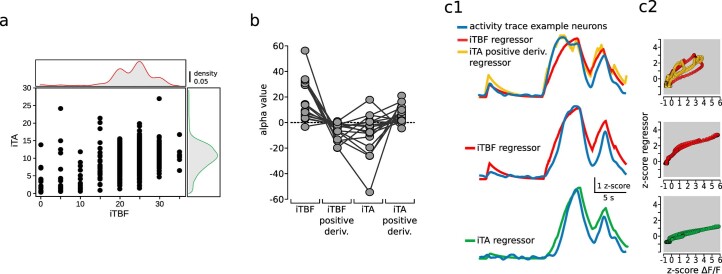
Modelling activity of medullary V2a RSNs as a function of kinematic parameters. **a**, Distribution and correlation between the different instantaneous swimming parameters. Instantaneous tail beat frequency and tail amplitude are co-dependent, but not linearly correlated. Distribution of the computed instantaneous tail beat frequency (red, top) and instantaneous tail amplitude (green, right) (2176 timesteps, in 4 fish, 8 trials). **b**, Correlation coefficient of each motor regressor tested in the multiple linear models and illustrated for one example plane in one fish. c, Single neurons are explained by different motor regressors. c1, Neural activity of single neurons shown in Fig. 8 overlaid with the motor regressor that best explains it. c2, For each timestep, the value of the motor regressor (x axis) is represented as a function of the mean activity of the forward cluster (y axis). Source data

We noticed that the calcium signals of these medullary V2a RSNs in the forward cluster increased throughout the forward episode (Fig. 8d), suggesting that they could encode the number of oscillations during forward locomotion. We found a strong correlation between the maximal amplitude of the calcium transient and the number of oscillations during a forward bout (Fig. 8g1) for many medial V2a RSNs located in the rostral and caudal medulla (Fig. 8g2). To investigate which other kinematic parameters, among instantaneous TBF (iTBF) and instantaneous TBA (iTBA), and their rise, best explain V2a RSN activity, we used a regression-based approach to model the activity of individual neurons (Fig. 8h,i, Methods and Extended Data Fig. 4). Notably, the activity of most medullary V2a RSNs in the forward cluster encoded the iTBF (Fig. 8j, left; neurons whose coefficient was above 0 and substantially relevant: *n* = 59 of 89 neurons; Methods). Interestingly, a group of V2a RSNs in the caudal medulla encoded both the iTBF and the number of oscillations (Fig. 8j, right, red and blue circles). In contrast, the activity of other V2a RSNs in the medial medulla encoded both the iTBF and the rise in TBA during forward swims (Fig. 8j, right, red and yellow circles). Altogether, our results highlight that medullary V2a RSNs have sustained activity during forward locomotion and mainly encode the instantaneous locomotor frequency. Remarkably, we found a striking similarity between the activity maps of medullary V2a RSNs obtained during spontaneous (Fig. 7) and MLR-induced forward locomotion (Fig. 8k), indicating that MLR stimulation recruits similar clusters of medullary V2a RSNs that are endogenously active for voluntary movements.

## Discussion

Our study provides the identification of the locus of MLR in larval zebrafish with functional, anatomical and molecular characteristics that are consistent to those previously described in other vertebrate species. Using an unbiased approach relying on whole-brain imaging, we confirm that the MLR locus contains a high density of motor-correlated neurons whose activity reflects the vigor of bouts independently of the sensory context. We demonstrate that glutamatergic MLR neurons project onto and recruit RSNs, but not the nMLF.

Description of the MLR in larval zebrafish enabled us to fully exploit its optical and genetic accessibility to understand at the whole-population level how specific RSNs are recruited to initiate and control forward locomotion. We focused on an essential population of RSNs expressing the transcription factor *vsx2*, which we therefore referred to as ‘V2a’ RSNs. We show that the MLR differentially projects to V2a RSNs throughout the hindbrain. While ventral P and RP V2a RSNs receive somatic projections from MLR neurons and are all recruited at low intensity, dP, RP and medullary V2a RSNs receive only dendritic projections and are gradually recruited on increasing intensity of the MLR stimulation. Using high-speed volumetric imaging during volitional locomotion, we built a full hindbrain locomotor map of V2a RSNs that are distributed along the hindbrain. By taking advantage of the exceptionally long forward swims induced by MLR stimulation, we showed that a previously overlooked group of medullary V2a RSNs maintain their activity during forward locomotion and encode essential parameters controlling the duration and speed of forward locomotion.

### Identification of the MLR in larval zebrafish

The MLR is located at the mesopontine border along the cholinergic nuclei spanning from the caudal border of the substantia nigra to the LC^9^. In mammals, the MLR consists of the PPN and CnF in the rostral mesopontine cholinergic stripe^10^. In salamanders and lampreys, the most efficient part of the MLR to induce locomotion is located in the caudal mesopontine cholinergic stripe corresponding to the LDT^36,37^. In zebrafish, we identified the MLR as a small region approximately 40 µm in diameter and containing approximately 190 neurons. The zebrafish MLR is medial and dorsal to the LC and extends rostrocaudally in the PP area. Like previous findings in lampreys and salamanders, the zebrafish MLR locus is highly heterogeneous and includes glutamatergic, GABAergic and cholinergic neurons, presumably corresponding to the LDT^32^. In addition, we showed evidence that MLR neurons receive dopaminergic modulation via the D_1_ receptor. As shown in other species, the MLR locus in zebrafish is defined by its ability to initiate graded forward locomotion, and this function is carried by glutamatergic neurons projecting onto RSNs.

Furthermore, we collected a large whole-brain imaging dataset of larval zebrafish performing forward locomotion either spontaneously or when visually stimulated by moving gradings. As shown in rodents, where the activity of a fraction of MLR neurons encoded locomotor speed^48,49^, the activity of a fraction of MLR neurons in zebrafish is motor-correlated and reflects the vigor of motor bouts. In addition, motor-correlated neurons in the zebrafish MLR locus are active both for volitional and visually evoked locomotion. These observations concur with an independent analysis of information flow on whole brain stem imaging data during OMR performed using an improved Granger causality pipeline^50^. Using this analysis, motor-correlated neurons in the MLR during OMR were found to drive the activity of contralateral MLR neurons and motor-correlated neurons in the downstream reticular formation.

### The nMLF is not the MLR

Our study identified the MLR locus in larval zebrafish, thereby correcting a longstanding assumption that the nMLF could correspond to the MLR^27,28^. Although stimulation of the nMLF in fish effectively induces locomotion^24–26,51–53^, we argue that the nMLF is not the MLR because it is not located within the mesopontine border and because it directly projects to the spinal cord^29^. The nMLF acts as a sensorimotor coordination center because it receives sensory inputs from the visual^54^ and vestibular systems^55^ and in turn controls eye and head–tail positioning^52,55^, and sends descending projections into the spinal cord^29^ to control spinal motor neurons^56^. Similar to our findings on the MLR locus, the activity of the nMLF correlates in zebrafish with spontaneous^52^, visually and vestibularly evoked locomotion^25,52,57^, possibly reflecting a role in the tight coordination between eye and tail movements. In zebrafish, the term nMLF was adopted in the first descriptions of the spinal-projecting neurons^29^. Nevertheless, the term nMLF was originally used as a synonym of a structure called the interstitial nucleus of Cajal (INC)^58^. The INC projects to the spinal cord^59^ and coordinates eye and head movements, assisting the body in adjusting posture^60,61^. Interestingly, INC stimulation in cats and rats can also elicit locomotion^62^. Based on its anatomical and functional properties, we propose that the homology between the nMLF and INC must be considered when drawing conclusions from comparative studies.

Remarkably, we found that the nMLF is not recruited on stimulation of the MLR, despite being at closer distance than most RSNs recruited from the stimulation electrode. Evidence that MLR neurons are active for spontaneous and visually evoked locomotion supports a model in which nMLF and MLR act as parallel pathways, triggering in a coordinated manner the initiation and steering of locomotion in combination with changes of posture.

### Control of graded forward locomotion

Animals move using different types of gaits. Quadrupedal mammals walk at low speeds, trot at intermediate speeds and gallop at high speeds. The MLR controls locomotor speed and its stimulation at different strengths evokes natural changes in gait^9^. For instance, stimulating the MLR in salamanders at low intensity produces walking (slow gait), but at higher stimulation intensity the animal transitions to swimming (faster gait)^36^. Larval zebrafish swims in a beat-and-glide fashion, where short periods of activity of typically few hundreds of a millisecond with locomotor frequencies ranging between 15 and 80 Hz^63^ are followed by long interbout intervals where the animal is not actively swimming. Evidence for two different forward gaits has been reported in freely swimming larval zebrafish during optomotor response as a function of grating speeds^25^ and in fictive preparations on skin stimulation^27^. The average locomotor frequency during slow swimming occurs between 15 and 30 Hz, while it ranges between 30 and 80 Hz during fast swimming^25^. MLR stimulation only triggered forward locomotion in the slow regime, occurring either at 17 Hz for stimulation frequencies below 15 Hz, or at 23 Hz for MLR stimulation at 20 Hz. Our MLR stimulations never produced locomotor frequencies above approximately 25 Hz that can be deployed for avoidance or escape. It is possible that our current definition of the MLR site based on the induction of forward locomotion may correspond to the homolog of the PPN in mammals^48^, which only produces low locomotor frequencies. We cannot exclude that stimulation of adjacent areas, homolog of the CnF, could trigger fast swimming. Future studies will elucidate this point.

### Anatomical and functional coupling of MLR neurons to V2a RSNs

In line with previous studies identifying the MLR^10^, using focal electroporation and retrograde tracing, we demonstrated that MLR neurons send commissural projections to the contralateral MLR, and bilateral descending projections to the reticular formation^64^. Experiments done in lampreys^5^, mice^17^ and cats^65^ showed that only a small proportion of RSNs are active during spontaneous or MLR-induced forward locomotion. In agreement with these observations, we find that the MLR recruits a low proportion of brain stem V2a RSNs. At low intensity, the zebrafish MLR recruits ventral V2a RSNs in the P and RP regions in an all-or-nothing fashion, consistent with previous reports of large excitatory postsynaptic potentials previously recorded in these cells^13,38,39^. This effective coupling can be explained by axosomatic contacts formed by MLR neurons onto P or RP V2a RSNs. In contrast, MLR stimulation gradually recruits the medial V2a RSNs in the medulla, similarly to what has been observed in lampreys and salamanders in unidentified RSNs^38,39^. This weak coupling can be explained by MLR neurons projecting onto the dendrites, and not the soma, of medullary V2a RSNs.

The present results clearly indicate that MLR neurons project to specific populations of RSNs distributed throughout the hindbrain, with the strongest coupling to P and RP RSNs. Note that in mammals, a large emphasis has been placed on medullary RSNs. Based on our findings, MLR inputs to P and RP RSNs should be further investigated in mammals.

### A conserved organization of ‘steering’ and ‘forward’ V2a RSNs

In mice, the activation of V2a RSNs in the rostral medulla triggers stops on bilateral^22^ activation and turns on unilateral stimulation^19,20^, while in zebrafish the global activation of V2a RSNs elicits forward locomotion^18^. This apparent discrepancy led to the assumption that the behavioral role of V2a neurons was not conserved between teleostean and mammalian species^3,22^.

By mapping the activity of virtually all hindbrain V2a neurons during steering and forward locomotion, our study refutes this proposal and points to a strong conservation of the locomotor roles of hindbrain V2a neurons in vertebrates. We found many large V2a RSNs in the P and RP regions that were recruited both for forward locomotion and steering to the ipsilateral side. This observation is consistent with reports from other species that steering movements are induced by asymmetrical activation of RSNs (lamprey^4^, salamander^66^ and rat^67^). Our decomposition analysis into motor components revealed that these ventral P and RP RSNs are symmetrically active for forward bouts and asymmetrically active during turns^57^. Unilateral ablation of a subset of ventral P and RP RSNs previously showed that these neurons contribute to the first tail bend^21^. Our results are consistent with early optogenetic investigations of the V2a population in young larval zebrafish (3 dpf), which had mentioned anecdotally that activation of rostral brain stem V2a neurons more often triggered turns (supplementary information of ref. ^18^).

In contrast to these V2a RSNs involved in both forward and steering, distinct V2a RSNs exclusively recruited for steering were localized in two clusters in the RP region (r6) and rostral medulla (r7). The location of these steering-selective V2a RSNs matches with the steering V2a RSNs in the caudal P reticular nuclei (r6) and rostral medulla (r7) in mice^19,20^ (see Fig. 1c for anatomical equivalences). Additionally, in the fish rostral medulla (r7), V2a RSNs encoding the forward component are intermingled with V2a RSNs encoding steering. This description is concordant with recent recordings in mice showing that V2a neurons in the rostral medulla form a heterogeneous population encoding steering or locomotor initiation^68^.

### Medullary V2a RSNs encode the duration and frequency of forward swimming

We found that a subset of medial V2a RSNs in the medulla (r7 and r8) are active during the forward component of swimming and sustain their activity during forward locomotion displayed on MLR stimulation. This is consistent with recruitment of medial V2a RSNs in the caudal medulla for weak short and forward fictive bouts^18,41^. Our work uncovers that medullary V2a RSNs in r8 encode instantaneous frequency and the number of oscillations probably operating as ‘maintain’ V2a RSNs. Consistently, the ablation of medial V2a RSNs in the caudal medulla of larval zebrafish resulted in shorter forward bouts^41^. By decomposing the behavior into motor components, we also showed that a subset of medial medullary V2a RSNs neurons encodes the forward oscillatory component of all bouts.

Most medullary V2a RSNs in larval zebrafish project a descending axon ipsilaterally^69^ and resemble medullary RSNs previously recorded in *Xenopus* tadpoles^70^, where these neurons spike in phase and precede each motor burst. We found in zebrafish that ‘maintain’ V2a RSNs are dense in the caudal-most part at r8 at the border with the spinal cord. Targeted electrical and optogenetic stimulation in this region robustly induced locomotion^71–73^, and this area has been found to generate the locomotor rhythm in isolation^74^. This converging evidence suggests that this area is a critical hub to gate information from brain stem to spinal cord, in which medullary V2a RSNs may act as a recurrent network triggering the oscillations of the spinal central pattern generators to sustain the forward locomotor rhythm. Noteworthily, this hindbrain region receives specific descending projections from the nMLF to induce forward locomotion at different speeds^26,75,76^. Therefore, we propose that the forward-component V2a RSNs located in the caudal medulla act as a hub integrating parallel descending commands from distinct brain areas, the MLR and nMLF, to start and maintain forward locomotion.

The caudal and medial medullary V2a RSNs, acting as ‘maintain’ cells for forward locomotion, could correspond to the glutamatergic V2a neurons located in the gigantocellular nuclei in mice^17,22^. Glutamatergic neurons from the same region receive axodendritic inputs from the MLR and elicit forward locomotion at different speeds^12^. An atlas of molecular markers across vertebrate species will be of interest to resolve the correspondence between nuclei described in mice and the groups of RSNs described in larval zebrafish in this study.

Altogether, our study identified the MLR in larval zebrafish and mapped the downstream command circuits involved in forward locomotion. Our work represents an essential step for future research on supraspinal motor control because virtually each and all RSNs can be uniquely identified, recorded and manipulated during active locomotion in this transparent and genetic model organism.

## Methods

### Animal care and transgenic lines

Animal handling and procedures were validated by the Paris Brain Institute (ICM) and the French National Ethics Committee (Comité National de Réflexion Éthique sur l’Expérimentation Animale; APAFIS no. 2018071217081175) in agreement with European Union legislation. To avoid pigmentation, all experiments were performed on *Danio* *rerio* larvae of AB background with the *mitfa*^−/−^ mutation. Adult zebrafish were reared at a maximum density of eight animals per liter in a 14/10 h light–dark cycle environment at 28.5 °C. Larval zebrafish were typically raised in Petri dishes filled with system water under the same conditions in terms of temperature and lighting as for adults. Transgenic larvae expressing Kaede and used for the photoconversion experiments were exceptionally raised in complete darkness to minimize global photoconversion at the early stages. Experiments were performed at 20 °C on animals aged between 4 and 7 dpf as described in each experimental protocol below.

The following transgenic lines were used: *Tg(vmat2:GFP)*^*zf710Tg*^^77^, *TgBAC(vsx2:GAL4FF)*^*nns18Tg*^^18^, *Tg(vsx2:Kaede)*^*nns2Tg*^^43^, *Tg(UAS:GCaMP6s)*^*mpn101Tg*^^52^, *TgBAC(slc17a6b:loxP-DsRed-loxP-GFP)*^*nns14*^ (ref. ^78^) referred to in the text as *Tg(vglut2:DsRed)*, *Tg(KalTA4u508)*^*u508Tg*^^40^, *Tg(UAS:jGCaMP7f)*^*u341Tg*^^40^, *Tg(UAS:ChrimsonR-tdTomato)*^*u328Tg*^^79^, *Tg(elavl3:GCaMP6f)*^*jf1Tg*^^30^, *Tg(UAS:kaede)*^*s1999t*^^80^.

### Design of the *Tg(KI-drd1ra:GAL4)* chimera and stable line *Tg(GAL4FF)*^*uot17*^

To generate the *Tg(KI-drd1ra:GAL4)* chimera, we inserted the *hps70:GAL4* cassette into the upstream locus of the *drd1ra* coding sequence according to the strategy from ref. ^34^ based on CRISPR–Cas9-mediated genome editing. In this approach, two short gRNAs were designed: one is IDT_1 (5′-TTTAAGACCAGTCACACCTC-3′) to target the insertion locus of *hps70:GAL4* cassette at −84 bp from the start codon of the *drd1ra* coding sequence in the genome; the other is Gbait (5′-GGCGAGGGCGATGCCACCTA-3′) to linearize the donor plasmid *Gbait-hsp70:Gal4FF* containing a Gbait (GGCGAGGGCGATGCCACCTACGG) sequence (which is derived from enhanced green fluorescent protein (GFP)) and a heat shock promoter hsp70 sequence^81^ followed by a GAL4 (GAL4FF) reporter^82^ and a bovine growth hormone poly(A) sequence^18^. The Cas9 mRNA was synthetized from a pCS2-SpCas9 plasmid. A total 5 mg of the plasmid was digested with BbsI (New England Biolabs); Cas9 mRNA was transcribed using the mMESSAGE mMACHINE T7 Ultra Kit (Thermo Fisher Scientific). Both sgRNAs and *Cas9* mRNA were coinjected into one-cell-stage zebrafish *Tg(UAS:Kaede)* embryos with fresh Xtra Midiprep (MACHEREY-NAGEL) purified *Gbait-hsp70:Gal4FF* donor plasmid. All embryos were injected with a 1-nl solution containing 7 ng ml^−1^ of each sgRNA, 150 ng ml^−1^ of *Cas9* mRNA and 7 ng ml^−1^ of donor plasmid. Fluorescent protein expression was monitored at 3–4 dpf; larvae showing fluorescence in the brain and spinal cord were selected for imaging.

*Tg(GAL4ff)*^*uot17*^ was generated using the same strategy using the *drd1a* gRNA 5′-TGACAACAGTCATTGACAGTGG-3′ predicted cut site 39 bp downstream of the *drd1a* start codon. To generate *Tg(UAS:mScarlet)*^*uot18*^, a codon-optimized *mScarlet*^83^ fragment was cloned into a *pTol2-UAS:MCS* plasmid and the resulting plasmid was used to generate this line.

### MLR electrical stimulation

Zebrafish 6–7-dpf larvae were embedded in 2.5% low melting point agarose (Thermo Fisher Scientific) in a 35-mm Petri dish. The dish was filled with external bath solution (134 mM NaCl, 2.9 mM KCl, 2.1 mM CaCl_2_-H_2_O, 1.2 mM MgCl_2_, 10 mM glucose and 10 mM HEPES, pH adjusted to 7.4 and osmolarity to 290 mOsm). The agarose was removed caudally to the swim bladder to leave the tail free. Homemade glass-coated tungsten microelectrodes (0.7–3.1 MΩ, 1–2-µm tip) were used to perform monopolar electrical stimulations. The electrodes positioned with a 35° angle penetrated the skin at different sites in the PP region using a motorized micromanipulator (MP-285A, Sutter Instruments). To bring the electrode in the MLR position, we used the LC as a landmark in the *Tg(vmat2:GFP)* transgenic larvae. Stimulation was delivered using an isolated stimulation unit (2100, A-M Systems) triggered by a Digidata series 1440A Digitizer (Axon Instruments, Molecular Devices) that synchronized electrical stimulations and behavioral recordings controlled by the Clampex v.10.3 software (Axon Instruments, Molecular Devices). For the initial search of the MLR, the behavioral response to stimulations in each locus was monitored by applying continuous stimulation at 10 Hz and 1 µA for less than 30 s; a rest period of at least 3 min was allowed between successive loci. After five tested loci without obvious observed behavioral response, the larva was discarded. Once the MLR locus was found, we implemented experimental trials lasting 1 min during which the stimulation protocol consisted of trains with 2-ms negative pulses, occurring at 5–20 Hz with 0.1–3 µA intensity lasting for 1–4 s. To allow recovery and avoid synaptic habituation, the resting period between trials was at least 2 min. For experiments on paralyzed *Tg(vsx2:GAL4;UAS:GCaMP6s)* transgenic zebrafish, the stimulation protocol consisted of single 2-ms negative pulses with increasing intensities (1–5 µA) in steps of 1 µA and intertrial intervals of 20 s. For the same fish, a rest period of at least 3 min was allowed between trials recorded for different imaging planes. All electrical stimulation experiments were carried out in a confocal microscope combining an upright microscope (Examiner Z1, ZEISS), using a ×20/1.0 differential interference contrast (DIC) D = 0.17 M27 75-mm objective (catalog no. 421452-9880-000, ZEISS), a spinning disk head (CSU-X1, Yokogawa) and a laser light stack (LaserStack, 3i Intelligent Imaging Innovations).

### Behavioral recording and analysis

#### Behavioral recording

Head-embedded larval zebrafish were illuminated from the side with a 45° angle using an 890 nm light-emitting diode (LED) (part no. ILH-IW01-85SL-SC211-WIR200, Intelligent LED Solutions) and behavior was recorded at 300 Hz from below through a ×5 ×20/0.25 12.5-mm microscope objective (part no: 440125-0000-000, ZEISS) and imaged on a high-speed camera (acA640-750um, Basler) with a 50-mm lens (catalog no. MVL50M23, Navitar). When the behavioral recording was coupled with functional calcium imaging, the 488-nm imaging laser was blocked with a 600-nm high-pass filter located in front of the camera. The high-speed camera was controlled using the Hiris software (RD Vision, https://www.rd-vision.com/r-d-vision-eng).

#### Tail tracking and automated segmentation of bouts

We used the open-source software ZebraZoom (https://github.com/oliviermirat/ZebraZoom) to track the position of the tail for each frame and extract bouts, defined as discrete events when the tail was continuously moving. The tail angle was defined as the angle between the body axis of the fish and the tip of the tail. The quality of tail tracking was carefully assessed by visual inspection. Electrically induced bouts were defined as all bouts starting during the electrical train stimulation. Swim bouts starting just before the stimulation were excluded from the analysis. ‘Spontaneous’ bouts were defined as bouts starting and ending before the first electrical stimulation of the trial. Note that bouts occurring in the 1-min time window after a stimulation train were excluded from the analysis because their properties could have been impacted by the previous electrical stimulation.

#### Segmentation of episodes within a bout

In the behavioral responses to electrical stimulations, bouts sometimes occurred as mixed episodes of forward swims and escapes or struggles. Therefore, we manually segmented all bouts into episodes of either forward swims or struggles. The procedure was blindly implemented by an investigator who ignored the electrode position and the stimulation protocol used. Three rules were followed to maximize consistency: (1) forward swims were defined by symmetric tail angle traces below 25° with a minimum of three oscillations; (2) long forward episodes with rare tail bends above 25° were kept as single forward episodes; (3) escape or struggle episodes were defined as the series of large bends (tail angle greater than 25°) assigned as one single episode, but segmented into multiple episodes when bends were separated by more than 100 ms.

#### Analysis of tail kinematics

From the tail angle, the ZebraZoom algorithm extracted the timing and amplitude of each tail bend that we then used to calculate iTBF and iTBA. For each episode, we estimated the corresponding duration, number of oscillations and maximum tail bending amplitude as previously done for bouts in freely swimming animals, together with the median iTBA and iTBF (https://github.com/wyartlab/Carbo-Tano_Lapoix_2023).

#### Definition of the behavioral forward index

To estimate the efficiency of a stimulation site to elicit forward swimming, we computed for each stimulation trial a forward index defined as

$$\begin{array}{l}{\rm{Forward}}\,{\rm{index}}\_{\rm{stim}}\\=({\rm{number}}\,{\rm{of}}\,{\rm{forward}}\; {\rm{swim}}\,{\rm{episodes}}-{\rm{number}}\,{\rm{of}}\,{\rm{escapes}}/{\rm{struggles}})/\\{\rm{number}}\; {\rm{of}}\; {\rm{all}}\; {\rm{episodes}}\end{array}$$

As we described in Results, electrical stimulation at frequencies lower or above 10 Hz, or with intensities above 2 µA, elicited mainly escape or struggle behaviors. For this reason, we computed the median forward index of each stimulation site (Fig. 2) only using the stimulation trials with optimal stimulation parameters, that is, trains of pulses at 10 Hz with current amplitudes of 1–2 µA.

### Whole brain light sheet functional imaging data

The data used for this section were previously collected and published^35^ (see ‘Functional whole brain imaging data analysis’ section in Methods). Briefly, neural activity and tail movement were recorded during optomotor behavior of 6–8-dpf-old *Tg(elavl3:GCaMP6s)* zebrafish larvae expressing the cytosolic calcium indicator GCaMP6s under a pan-neuronal promoter. During the experiment, head-restrained larvae were presented with a grating moving in a caudal to rostral direction at a speed of 10 mm s^−1^, while a high-speed camera was tracking the tail. Neural activity was recorded at 2 Hz (two whole-brain volumes per second) for 1 h. Each trial consisted of a stimulus period of 15 s preceded and followed by 7.5-s periods of static grating. Experiments were performed in a closed loop. Data were registered to a Z-stack of larval zebrafish reference brain previously obtained by coregistration of 23 confocal Z-stacks of zebrafish brains with pan-neuronal expression of GCaMP6f (*Tg(elavl3:GCaMP6f)*). The MLR region was then defined on this reference brain. Regions of interest (ROIs) within the mask were extracted for subsequent analyses.

#### Functional whole brain imaging data analysis

ROIs were extracted from the imaged volumes via a segmentation process based on the pixel-wise correlation map. First, each pixel was assigned a value that was the correlation between the fluorescence time trace of that pixel and the average trace of eight adjacent pixels in the same plane. Second, based on the correlation map, individual ROIs were segmented by growing from each high-intensity pixel and assigning neighboring pixels with a minimum correlation exceeding a certain threshold. The threshold was set at 0.3 for the closest neighbor to the seed and increased linearly to 0.35 at a 3-μm distance. The process started with the pixel with the highest correlation in the map as the first seed. Each time an ROI was set, a new seed was considered as the highest correlation pixel remaining with correlation higher than 0.3. To ensure that ROIs had approximately the size of somata, their area was restricted to the range of 9–28 μm^2^. Finally, the fluorescence time trace of each ROI was extracted as the sum fluorescence signals of all pixels that were assigned to that ROI during segmentation.

Fluorescence traces from ROIs within the MLR were detrended to remove linear drifts and *z*-scored. Recorded tail traces were also *z*-scored. Swim vigor and swim bouts were extracted using the bouter package (https://portugueslab.github.io/bouter/index.html). Briefly, vigor was estimated as the sliding standard deviation of the tail trace with a time window of 50 ms. Swim bouts were then automatically detected by thresholding the vigor trace. Three types of regressors were constructed for the correlation analysis: a stimulus regressor, a motor regressor and a vigor regressor. These were obtained by, respectively, convolving the on/off stimulus trace, on/off bout trace and the vigor trace with an exponential kernel using the decay time constant of GCaMP6s. These convolved traces were then downsampled to the functional imaging frequency to get the actual regressors. Finally, we calculated the Pearson correlation between each regressor and the fluorescence traces from the MLR.

We then defined MLR neurons as correlated with motor activity (Fig. 2l), if the correlation value to motor activity was above a threshold defined as the 75th percentile of motor correlation values of all ROIs recorded (threshold defined independently for each fish). With this approach, we aimed to give an overview of how many MLR neurons were more motor-correlated than the rest of the neurons. Then, we aimed to specifically look at which MLR neurons could be responsible for eliciting and controlling the locomotor output. We selected ROIs, within the MLR, whose correlation to motor activity was higher than the sensory stimulus, and whose correlation to vigor of the bout was within the 25th top percentile of vigor correlation values within the MLR (depicted as vigor-correlated in Fig. 2k; examples trace in Fig. 2m). These specific MLR neurons were used to explore the link between calcium activity and vigor during spontaneous or visually induced locomotion (Fig. 2n).

### Anatomical investigations

#### Registration of electrical stimulation sites in the brain atlas

To assess the position of the stimulation electrode, Z projection confocal stacks were acquired combining the GFP signal and the DIC channel using a ×20/1.0 DIC D = 0.17 M27 75 mm objective. The Z-stacks were acquired with a step of 1-µm depth using the Slidebook software v.6.0 (3i Intelligent Imaging Innovations). From each image volume, we annotated manually in Fiji (https://fiji.sc/Fiji) the X, Y and Z coordinates of the electrode position from the DIC channel and three anatomical reference coordinates from the GFP pattern of expression of the *Tg(vmat2:GFP)* transgenic line (medial boundary of the LC on each side, anterior limit of the superior raphe and anterior limit of the inferior raphe). Using a custom-written Python script, the coordinates of the anatomical landmarks were transformed to the mapzebrain fish space (https://mapzebrain.org/home) for the *Tg(vmat2:GFP)* transgenic line and the resulting transformation matrix was then applied to the electrode coordinates.

#### Focal electroporation and neuronal tracing

Focal electroporation was performed according to standard protocols^44^. Briefly, 6-dpf larvae were anesthetized with tricain (MS-222, 0.02%, Sigma-Aldrich) and mounted on a 35-mm Petri dish. A small portion of the agarose was removed on top of the midbrain–hindbrain boundary. A micropipette was filled with a solution of fluorescent dextran (Dextran, Alexa Fluor 647, 10,000 MW, fixable, 0.2 mg ml^−1^ in distilled water, catalog no. D22914, Thermo Fisher Scientific) and cells were electroporated in the MLR locus. A Grass S88 stimulator (Grass Technologies) was used to deliver two trains of voltage pulses. Each train consisted of 2-ms pulses with an amplitude of 4 V delivered at 200 Hz for 250 ms. After the procedure, larvae were unmounted and allowed to recover for 3 h. Larvae were then anesthetized, dorsally mounted in 1.5% low-melting agarose and imaged using a FV1200 Olympus scanning confocal microscopy. The acquired brain volumes were registered to the mapzebrain reference brain using the landmark registration function of 3D Slicer (v.5.0.2 r30822/a4420c3). Axonal projections were traced using the Simple Neurite Tracer plugin for Fiji.

#### Retrograde labeling experiments using biocytin

For the retrograde labeling experiments, 6-dpf larvae were anesthetized with 0.02% MS-222 (catalog no. A5040, Sigma-Aldrich) and embedded dorsally in 1.5% low melting point agarose prepared in external solution (as described above). A small portion of the agarose was removed on top of the hindbrain and all the liquid was then extracted from the dish. A few crystals of biocytin Alexa Fluor 594 (catalog no. A12922, Thermo Fisher Scientific) were diluted in 5 µl distilled water and then allowed to recrystallize on the tip of insect pins (stainless steel Minutiens, 0.2-mm diameter, Austerlitz). Under a stereo dissecting microscope, a unilateral single lesion in the hindbrain between r2 and r6 was done using the dye-soaked insect pins. After the procedure, the dish was filled with external solution, the larvae were unmounted and allowed to recover for 2 h in oxygenated external solution. Larvae were then again anesthetized with 0.02% MS-222 and mounted in 1.5% low melting point agarose. The retrogradely labeled neurons were imaged in a confocal microscope as described above. From each image volume, the coordinates of the backfilled neurons in the region of the MLR were annotated using Fiji. The positive backfilled neurons selected for this analysis were taken from the MLR area defined by electrical stimulations (with a diameter of approximately 40 µm). For the experiment in Extended Data Fig. 1a, the injections were done in wild-type fish. As the dye injection forms a background labeling in the entire nervous system, we chose four anatomical landmarks also easily distinguishable in the mapzebrain 4,6-diamidino-2-phenylindole fish space. For the experiments in Extended Data Fig. 1c, the four anatomical landmarks were selected on the basis of the DsRed fluorescence pattern in each transgenic *Tg(vglut2:DsRed)* larva and from the mapzebrain *Tg(vglut2a:DsRed)* fish space. The script used and the registration steps applied were the same as described in the previous section.

#### Optical backfills of RSNs by spinal photoconversion of Kaede

Transgenic *Tg(vsx2:Kaede) z*ebrafish larvae expressing Kaede protein in V2a neurons were raised in darkness. At 4 dpf, larval zebrafish were anesthetized in 0.02% MS-222 and mounted dorsal side up in 1.5% low melting point agarose. The Kaede protein in the rostral spinal cord (segments 4–9) was photoconverted by using an ultraviolet LED (405 nm, part no. SOLIS-405C, Thorlabs) during a 2-min illumination sequence (500 ms ON and 500 ms OFF pulsed illumination, repeated twice) through a ×40 NA = 0.8 water immersion objective (power: 1.02 mW mm^−^^2^). This photoconversion procedure was repeated five times with intervals of 40 min. Digital mirror devices were used to pattern light on spinal segments 4–9. On completion, larvae were kept in the dark for 8–12 h to allow the photoconverted Kaede protein in the rostral spinal cord to travel back up to the hindbrain to label the soma of spinal-projecting V2a neurons. The larvae were next imaged using a confocal microscope with a spinning disk head (CSU-W1, Yokogawa) on an upright microscope (Examiner Z1, ZEISS), using the 488 nm and 561 nm laser light sources (LaserStack). Images were acquired using a ×20 NA = 1.0 DIC D = 0.17 M27 75 mm, using Slidebook v.6.0 (3i, Intelligent Imaging Innovations) and processed using Fiji. Given the optical accessibility of the larval zebrafish and our noninvasive and nonbiased approach, we assume, in our following calculation, that all RSNs are labeled by the optical backfill. Indeed, with a reliable genetic targeting, optical backfill shows high precision and much less variation than those observed after chemical backfill, which depends on many factors, such as the location of the lesion, the size of the axons sectioned and the size of the dye used.

#### Immunohistochemistry

For immunohistochemistry, 6-dpf larvae were processed as described above to label MLR neurons projecting to the reticular formation. After the injection, larvae were allowed to recover for 2 h in oxygenated external solution. Larvae were then euthanized using 0.2% MS-222 and fixed in paraformaldehyde 4% in PBS for 2 h at room temperature and then washed three times for 30 min in 1× PBS. The whole larvae were then transferred into a solution of 20% sucrose overnight at 4 °C. The next day, they were quickly frozen in methylbutane cooled to −45 °C and the heads were cut in a cryostat at 25-µm thickness in the transverse plane. The sections were collected in PBS containing 0.1% Triton X-100 (PBST, 0.1 M, pH 7.4, 0.9% NaCl) and then blocked with PBST containing 10% normal donkey serum (catalog no. 017-000-121, Jackson Immunoresearch) for 60 min at room temperature. The sections were then incubated for 48 h at 4 °C in PBST containing the following primary antibodies: rabbit anti-DBH (research resource identifier (RRID): AB_572229, diluted 1:400, catalog no. 22806, Immunostar) and goat anti-choline acetyltransferase (RRID: AB_2079751, diluted 1:100, catalog no. AB144P, Merck Millipore). Sections were then rinsed three times for 10 min in PBST and incubated for 4 h in PBST containing the following secondary antibodies: donkey anti-rabbit conjugated to Alexa Fluor 350 (diluted 1:200, catalog no. A10039, Invitrogen) and donkey anti-goat conjugated to Alexa Fluor 594 (diluted 1:400, catalog no. A11058, Invitrogen). Sections were then rinsed three times for 10 min in PBS, mounted on ColorFrost Plus microscope slides (Thermo Fisher Scientific) and coverslipped using VECTASHIELD (catalog no. H-1000, Vector Laboratories) as mounting medium. Sections were then observed and photographed using an E600 epifluorescence microscope equipped with a DXM1200 digital camera (Nikon). Grayscale images were converted to pseudocolors using Photoshop (v.23.1.1, Adobe). The primary antibodies used in combination here were tested separately resulting in similar labeling of the same neuronal structures. Removing the primary antibodies from the procedure, while keeping everything else the same, did not produce any labeling of neuronal structures in our material. The rabbit anti-DBH from this study was successfully used in zebrafish by other authors to label LC neurons specifically^84,85^. In our material, only LC neurons were labeled with this antibody. Alternatively, the rabbit anti-DBH from this study was replaced with a rabbit anti-tyrosine hydroxylase (RRID: AB_390204, diluted 1:400, catalog no. AB152, Merck Millipore). The resulting labeling of LC neurons was similar in every aspect to the one obtained with the DBH antibody. The goat anti-ChAT used in this study has been used with success on a wide range of vertebrate species, including zebrafish, to label cholinergic neurons^32,85,86^.

### Single-plane calcium imaging experiments using spinning disk microscopy

For in vivo recording of calcium activity in RSNs, we screened the brightest fluorescent *Tg(KalTA4u508;UAS:GCaMP6f) or Tg(vsx2:GAL4,UAS:GCaMP6s)* transgenic larvae at 3 dpf. On the day of the experiments, 6-dpf larvae were dorsally mounted as described above, with or without the tail left free to move depending on the experiments. For the experiments relying on MLR stimulations via single pulses, larvae were first paralyzed by bath application of 1 mM α-Bungarotoxin (Tocris) diluted E3 medium for 3–6 min. Calcium imaging activity was recorded using a confocal spinning microscope on an upright microscope (Examiner Z1; LaserStack). Images were acquired using a ×20 NA = 1.0 objective, using Slidebook v.6.0. Spinning disk time series were acquired at either 16 Hz (Fig. 5) or 10 Hz (Fig. 8) with a field of view (FOV) of 340 × 340 μm (pixel size = 0.66 μm). We chose to record planes representative of the different anatomical regions we imaged from, corresponding to planes at depths of 182, 225, 257 and 288 µm in the mapzebrain brain atlas. After imaging calcium activity in each plane, we systematically recorded an anatomical Z-stack of the entire hindbrain to localize the site of stimulation.

#### Single-plane calcium imaging data analysis

##### Preprocessing of time series

For all analyses of the fluorescence movies, we used the suite2p^87^ (v.0.8.0, https://github.com/MouseLand/suite2p). The pipeline first corrected the movies for motion artifacts in 2D using rigid and nonrigid registration. Most ROIs were automatically identified by the pipeline, and we manually added ROIs on cells that were not detected, including the soma of neurons that did and did not show activity during recording. Then, using a custom-built Python script, for each ROI: (1) we corrected the raw fluorescence trace by subtracting the neuropil signal (that is, the sum of surrounding pixels’ fluorescence, with a factor *n* = 0.7); (2) we calculated the *ΔF/F*(t) = (F(t) − F0) / F0, where F(t) is the fluorescence at time t and F0 is the cell baseline fluorescence (median fluorescence value selected on a 3-s period of inactivity); (3) we estimated the noise as the standard deviation of the signal during the same 3-s period of inactivity, which was further used to set the recruitment threshold of the cell; (4) the *ΔF/F* was filtered using an average running filter with three time steps. For visualization purposes and regression analysis, a low-pass filter was applied instead. In the tail-free experiments, when the motion artifact was larger and the 2D registration was not satisfactory, we removed the artifactual values by adding nonexistent values for frames in which the correlation to the mean image was low (we used the three lowest percentiles of correlation in the suite2p ops[‘corrXY’] output). To run the correlation and regression analyses, we linearly interpolated the missing values to process continuous time series. Neurons were assigned to the anatomical groups defined in Fig. 1 based on their rostrocaudal position in the hindbrain and the manually assigned boundaries between P, RP and medullary regions. Each neuron was assigned to either the ventral or dorsal group based on its normalized depth location in the mapzebrain atlas, using a dorsoventral limit depth of 100 µm for the P and RP regions and 80 µm for the medulla.

##### Registration of different planes across experiments

To compare the recruitment of V2a neurons across fish, we registered ROIs from the calcium imaging data of all fish to a shared brain space (corresponding to one reference fish illustrated in Figs. 5 and 8), as such: (1) anatomical boundaries, including the midline and the boundary between the medulla and RP region between r6 and r7, were visually identified, and shifted to match the boundaries of the reference fish; (2) the same shift was then applied to the position of each ROI to extract a normalized X and Y position in the reference fish; (3) we estimated for each plane its normalized depth by manually comparing to which depth the plane corresponded the most to the mapzebrain *Tg(vsx2:GAL4)* fish space. The normalized coordinates were subsequently used for mapping neurons position in 3D.

##### Recruitment analysis in paralyzed transgenic larvae

To define whether a cell was recruited after an electrical stimulation in Figs. 4 and 5, we tested whether its calcium trace significantly increased above baseline. To do so, we checked whether the maximal *ΔF/F* value between the stimulation start and 3 s after was above an arbitrary threshold defined as the baseline plus three times the noise (standard deviation of the signal during baseline). The baseline, for each cell at each stimulation, was defined as the mean signal over 1.5 s before the stimulation started. We defined ‘unresponsive cells’ as cells whose calcium trace did not reach the threshold in the 3 s for any stimulation. Reliably responding cells were the ones that reached the recruitment threshold systematically once a stimulation intensity was reached. Cells were classified as not reliably responding to the stimulation otherwise. For all cells that exhibited a calcium transient in response to the MLR stimulation, we computed the rising slope of the calcium trace as

$${\mathrm{rising}\;{\mathrm{slope}}}=\frac{{\frac{\varDelta F}{F}}_{{\mathrm{peak}}}-{\frac{\varDelta F}{F}}_{{\mathrm{onset}}}}{{t}_{{\mathrm{peak}}}-{t}_{{\mathrm{onset}}}}$$

in which *t*_onset_ is the time when *ΔF/F* starts rising after the stimulation and *t*_peak_ is the time where the calcium transient peaks. As filtering calcium traces would induce a lag in the signal, we computed these time points on the raw *ΔF/F*. These time points were semiautomatically defined for all exhibited responses.

##### Recruitment analysis in tail-free larvae

We investigated which V2a neurons were specifically recruited during forward swimming episodes by comparing their activity during forward versus struggle episodes. To do so, we extracted the maximum *ΔF/F* between 0.5 s before the start and 2 s after the end of the episode. As the max *ΔF/F* during a given episode could be influenced by the residual increase from a previous swimming episode, we corrected the max *ΔF/F* value by subtracting a normalized baseline (median *ΔF/F* of the signal over the 300 ms preceding the beginning of the episode). Under these conditions, a cell was recruited if its corrected maximum *ΔF/F* reached five times the noise (defined as above in the preprocessing section).

##### Forward activity index and identification of a forward cluster based on calcium activity

To identify neurons recruited during forward episodes elicited by MLR stimulation, we clustered neurons using calcium activity with an agglomerative clustering based on the Python package AgglomerativeClustering from the scipy.cluster library. We manually selected an arbitrary number of clusters (3–6) such that at least one cluster was specifically recruited during forward swimming only. To quantify that a group of neurons was indeed more active during forward swims, we computed for each neuron a forward activity index as

$${\mathrm{forward}}\;{\mathrm{activity}}\;{\mathrm{index}}=\frac{{\mathrm{mea}{n}}_{\frac{\varDelta F}{F}\left[{\mathrm{forward}}\;{\mathrm{swims}}\right]}-{\mathrm{mea}{n}}_{\frac{\varDelta F}{F}\left[{\mathrm{struggles}}\right]}}{{\mathrm{mea}{n}}_{\frac{\varDelta F}{F}\left[{\mathrm{forward}}\;{\mathrm{swims}}\right]}+{\mathrm{mea}{n}}_{\frac{\varDelta F}{F}\left[{\mathrm{struggles}}\right]}}$$

For each trial, we verified that the cluster of neurons assigned as ‘forward’ had the highest forward activity index.

#### Analysis of calcium signals for single-plane imaging

##### Linear regression between maximum *ΔF/F* and the number of oscillations

We used the package linregress from scipy.stats to model the maximum *ΔF/F* using the natural log of the number of oscillations. We used a logarithmic transformation to diminish the effects of the outliers in the distribution of the number of oscillations.

##### Motor regressors

To compare neuron’s activity to motor activity, we built multiple motor regressors defined as fictive calcium traces modeling distinct kinematic parameters. Specifically, we first built four time series describing TBA and TBF, and for each of them, the binary value (0,1) based on their positive differential reflecting their increase over time. These were built as follow: instantaneous tail beat amplitude: we resampled the tail angle trace (acquired at 300 Hz) to the corresponding sampling rate of calcium imaging (10 Hz), taking for each calcium imaging frame the maximal (in absolute) tail angle value; iTBF: we calculated the mean TBF of all the cycles happening during each calcium imaging frame; binary value of the positive derivative of the kinematic variables: for each parameter computed above, we computed a binary variable equal to 1 when the parameter was increasing over time and equal to 0 the rest of the time. To do so, we used a Heaviside step function on the derivative of the kinematic parameter after filtering with a running average filter using five frames.We then built the motor regressor convolving these time series with a GCaMP6s kernel with a negative exponential time decay of 1.5 s.

##### Multiple linear regression on calcium traces using motor regressors

As each neuron could drive multiple kinematic parameters, we examined whether calcium activity could be explained by the four motor regressors described above, using multiple linear regression to model each neuron’s calcium trace *ΔF/F*, at each time step *t*, as

$$\frac{\varDelta F}{F}\left(t\right)={\alpha }_{0}+{\alpha }_{1}{x}_{1}\left(t\right)+{\alpha }_{2}{x}_{2}\left(t\right)+{\alpha }_{3}{x}_{3}\left(t\right)+{\alpha }_{4}{x}_{4}\left(t\right)$$

with *x*1 the regressor for the TBA; *x*2 the regressor for the binary value of the increase in TBA; *x*3 the regressor for the TBF; and *x*4 the regressor for the binary value of the increase in TBF.

To fit the model on the data, we used the *z*-score of each kinematic parameter. We used a linear model optimized using ordinary least squares. The model aims to predict calcium activity using a linear combination of the coupling coefficients (*α*1, *α*2, *α*3, *α*4) minimizing the error between the data and the model. We performed permutation tests to assess the significance of a kinematic parameter in predicting the *ΔF/F* values (for significance, *α* needed to be positive and *P* < 0.05). Overall, the kinematic parameters significantly explained respectively: iTBF, 59 of 89 neurons; iTBA, 17 of 89 neurons; positive derivative iTBF, 6 of 89 neurons; positive derivative iTBA, 32 of 89 neurons.

### High-speed volumetric imaging using the SCAPE microscope

#### Conception of the oblique light sheet microscope (SCAPE)

The SCAPE microscope was built almost identically to the second version of the SCAPE microscope issued in 2019^46^. We added a few components to: (1) hold the sample and automate its translation; (2) record the tail movement; and (3) build a transmitted light image of the larval brain to be able to insert pipettes or electrodes in the brain to manipulate neural circuits. A 488-nm collimated laser was used to illuminate the sample (Coherent, OBIS 1220123), whose intensity is controlled by a filter wheel built with a range of neutral densities (catalog no. FW212CNEB, Thorlabs). The laser beam is shaped from a Gaussian beam to a homogeneous extended line using a Powell lens (Laser Line LOCP-8.9R30-1.0). The illumination light hits a 1D Galvo system (Gversus011/M 1D Large Beam (10 mm) Diameter Galvo System, Thorlabs) before going through an illumination telescope and at the back focal plane of the objective (1-U2B965 XLUMPlanFL ×20/0.95 W objective lens, Olympus). The galvanometer system scans the imaging plane by rotating the beam in the back focal plane of the objective. The laser beam is decentered from the center of the back focal plane of the objective to create the tilted light sheet. The objective illuminates the sample with a thin titled light sheet and collects fluorescence photons in the orthogonal direction. The emitted light is descanned and magnified by a detection system built of two telescopes, and corrects the light sheet angle using a system of two objectives (catalog no. MRD00205, CFI Plan Apochromat Lambda ×20/0.75 NA/1.0 mm attached to the detection telescope, and catalog no. MRD00105, CFI Plan Apochromat Lambda ×10/0.45 NA/4.0 mm attached to the camera system, both Morrel) mounted in a titled geometry to form a 2D image on a complementary metal–oxide–semiconductor sensor (Zyla sCMOS Zyla-5.5-CL10, Andor) orthogonal to the light beam propagation. A DAQ board controls the communication between electronic devices and the computer (NI USB-6361, National Instruments). To this initial setup, we added a sample holder (catalog no. MLS203P2, Thorlabs), an xyz motorized translation (catalog no. PT3/M-Z8, Thorlabs) controlled by KDC components (catalog no. KDC101, Thorlabs) to hold and move the 3.5-cm glass bottom Petri dish where the zebrafish larvae were placed. We also added components below the sample holder to record tail movement that are further described in the behavior recording and analysis section. The camera was controlled by the Andor Solis software, which was interfaced with a MATLAB program written by the Hillman lab to control the acquisition.

#### System calibration

To compute the pixel size in each axis, we followed the process indicated by the Hillman lab. For each given axis (*x*, *y*, *z*), we proceeded as follow: we imaged a volume of a sample of fluorescent beads (FluoSpheres 505/515, 1 µm, catalog no. F8888, Invitrogen). Then, we moved the sample of 0.1 mm along the given axis. We imaged the sample again after translation. We repeated this process to get the same sample of beads at ten given positions along the desired axis. Then, we measured the average displacement of each bead across these iterations. The pixel size was given as the mean, for all beads imaged, of the mean displacement of the bead across images, in pixels / mean displacement of the sample across images, in mm. The calibration parameters we measured corresponded to the ones of the Hillman lab system (*y*: 1.4 µm per pixel, *z*: 1.1 µm per pixel). However, the depth of field, corresponding to the maximum axial range with the optimal resolution, was smaller by a factor of 2 in our system than described in ref. ^46^. The extension of the FOV where the image is resolved depends on how well aligned the image plane and the camera plane are. The alignment is adjusted by moving the camera block or the height of the camera. This step is particularly challenging because these components are heavy and hard to move with the required precision. One solution we found was to add an extension to the support breadboard, but we did not manage to further improve the size of the FOV resolved.

#### Development of an open-source pipeline for deconvolution, motion correction and reshaping of the volumetric images

Once we had a functioning and aligned system, we aimed to build an analysis pipeline to extract from the recordings the positions and fluorescence signals of neurons monitored during the experiment. Briefly, the output images of an experiment, as acquired with the software from the Hillman lab, are *N*(t) 3D images of dimension (L_z_, L_x_, L_y_), where *N*(t) is the number of time steps. We combined the functions given by the Hillman lab, the open-source algorithms and homemade transformation scripts to transform these images into meaningful fluorescence traces.

##### Deconvolution with PSF

Imaging green fluorophores like GCaMP with 1 photon illumination requires using a laser around 488 nm, which is particularly prone to light scattering in tissues compared to higher wavelength. This issue leads to (1) loss of resolution, that is, the ability to distinguish two objects in space, which can prevent a proper segmentation of the image when trying to detect the position of the neurons in the image; (2) contamination of neurons’ fluorescence to the surrounding pixels, which creates ‘parasite’ signals in other neuron traces, that is, a signal that does not come from the neuron itself but from neighboring fluorescent structures (in our recordings, mostly surrounding the somas or axons of neurons). This phenomenon artificially increases the level of correlation between neurons’ signals and can prevail in a fine resolution of differences and similarities between neurons’ true signals. This problem is directly related to how much the image of an object spreads in space, which corresponds to the optical response of the microscope. Using the function that defines the spatial extension of the image of a single point in space, named point spread function (PSF), it is theoretically possible to restore the image of the original object via deconvolution. In practice, the process is only limited by imaging noise. With deconvolution, we aimed to retrieve the exact object light distribution by using a priori knowledge of the system. We proceeded as follow: first, we measured an experimental PSF, representing the impulse response of the optical microscope. We imaged multiple fluorescent beads smaller than the resolution of the microscope in a volume of similar size as the larval zebrafish hindbrain, computing the PSF of all beads, and then building a mean PSF from all measured PSFs. Then, each uncorrected functional volume of dimension (L_z_, L_x_, L_y_) was deconvolved with the Richardson–Lucy algorithm (RL) as implemented in the IOCBIO/deconvolve library (https://gitlab.com/iocbio/deconvolve). The RL deconvolution algorithm is well known and widely applied in biological imaging^88^. It follows an iterative maximum-likelihood approach and assumes that the noise is Poisson-distributed. Each pixel in an image deconvolved with RL is granted to be nonnegative. The RL algorithm needs the specification of only one parameter: the number of iterations, which needs to be set heuristically to enhance image quality yet avoid noise amplification artifacts^89^. The images presented in the manuscript were treated to obtain a Poisson noise fully compliant image and deconvolved with 15 iterations of the RL algorithm. On one central processing unit (CPU) (Intel Xeon(R) Silver 4214R CPU at 2.40 GHz), the deconvolution of a file of dimension (L_x_: 130, L_y_: 510, L_z_: 279 pixels, or approximately 34 MB) took approximately 91 s to complete.

**Extended Data Fig. 5:**
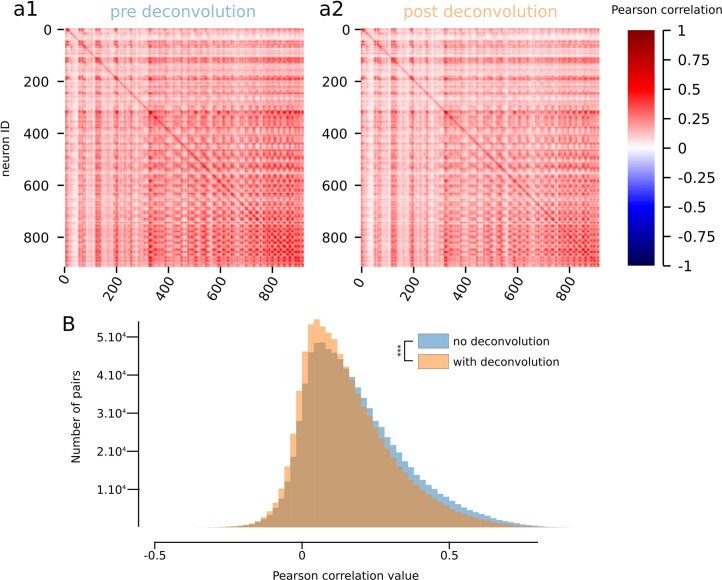
Effect of deconvolution on the correlation between V2a neurons’ signals recorded in the brainstem and spinal cord of one example fish. **a**, Pearson correlation matrix between the fluorescence signals of all pairs of neurons in an example recording, before (a1) and after (a2) deconvolution (n = 918 neurons, 1 fish). **a**, single-pixel value in the image represents the Pearson correlation coefficient between neuron at position x and neuron at position y. **b**, Distribution of the correlation values before (blue) and after (orange) deconvolution in an example experiment (n = 918 neurons, 1 fish, mean of absolute correlation values ± SD: no deconvolution 0.19 ± 0.16, with deconvolution: 0.17 ± 0.14).

The deconvolution significantly improved the contrast in the images and sharpened the borders between neurons, which became much easier to distinguish visually and were better detected by our segmentation algorithm (see four-plane wise segmentation, signal extraction and spike rate deconvolution using suite2p). We asked whether the deconvolution was also effective at reducing the levels of correlation between neurons’ fluorescence signals. Using the same pixel masks, we extracted the fluorescent traces of neurons with and without the deconvolution step with the PSF. We found that the correlation values were overall lower and more centered around zero when deconvolution was applied (Extended data Fig. 5). Altogether, these results show that deconvolution of functional calcium imaging data using experimental PSF is efficient at improving contrast in the image, improving segmentation performances and reducing signal contamination between surrounding neurons, therefore reducing correlations between fluorescent signals.

##### 3D motion correction using ANTsPy

In head-embedded, tail-free preparations, the larval zebrafish head is never completely stable. Indeed, large tail movements can create brain movement, resulting in motion artifacts in the recordings. Such motion artifacts are even more likely in head-embedded preparations because the animal reacts to the agarose surrounding its head by performing large tail bending resembling escape or struggle behavior. This is a problem because a neuron’s signal is extracted from the intensity of a fixed set of pixels in the image; if the neuron changes positions, we lose its signal during the motion artifact. One can compensate for such motion artifacts by transforming the image at which the motion happened until it looks the most similar to a reference image, a process called registration. To perform registration, we used a 3D registration library in ANTsPy (https://github.com/ANTsX/ANTsPy). The major source of motion artifacts in our recordings comes from brain movement when the fish contracts its musculature in the head or tail, during which the brain can change volume and the resulting brain image gets warped. Therefore, we chose to apply a nonrigid, deformable transformation, which gave better results than rigid or affine transformations. We registered the images in the imaging referential (before skew correction) for the same optimization reasons explained in the deconvolution section. We took the mean of the images in a whole video as a template and we registered each image against it. The mean image was chosen for its cost-effective calculation, low signal to noise ratio and robustness to the fluorescence fluctuation. Then, we applied affine and deformable registration (referred to as SyN) with default parameters. The typical processing time on one CPU for a file of dimension (L_x_: 130, L_y_: 510, L_z_: 279 pixels) was around 2 min.

##### Skew correction

Until this step, the images were processed in the SCAPE referential with dimension (L_z_, L_x_, L_y_). These images needed to be corrected for the angle at which the sample was scanned due to the tilted light sheet geometry to reconstruct the volume into a regular referential with dimension (L_z_, L_x_, L_y_). This step was performed using a MATLAB script adapted from the Hillman lab with an angle of correction of −47°. This angle was computed theoretically (from the distance of the center of the objective’s back aperture) and measured experimentally during SCAPE calibration. At the end of this step, we had *N*(t) volumes of shape (L_z_, L_x_, L_y_).

##### Plane wise segmentation, signal extraction and spike rate deconvolution using suite2p

To segment the image to detect the position of neurons and extract their fluorescence signal over time, we used the open-source, Python-based algorithm suite2p. This algorithm was developed for functional 2D images of calcium imaging recording. We decided to use this algorithm by decomposing our volumes into single planes. To decrease the probability of repeating the detection of the same cell in multiple planes, we extracted planes separated by 7 µm (the computed average diameter of V2a neurons’ soma in our recordings). We performed segmentation and signal extraction and successfully detected around 95% of the visually identifiable neurons.

We reshaped the *N*(t) acquired volumetric images to obtain Nz stacks of images. Each stack represents a movie of a given sample plane over time. We then used the open-source software suite2p to segment the image into ROIs, extract their pixel masks and their fluorescence signals. We used anatomical detection of the ROIs adapted from cellpose^90^, with the parameter diameter of ROIs as 6 or 7 pixels. After segmentation and mask creation, ROI fluorescence was extracted as the sum of the fluorescence of all pixels within the mask. For each ROI, we then corrected the fluorescence trace by subtracting the neuropil signal (with a factor *n* = 0.7). We calculated the *ΔF/F* of each cell as (F-F0) / F0, where F0 is the baseline signal calculated as the median fluorescence value on the lowest 10th percentile value. We removed remaining motion artifacts manually identified by masking them and, when needed, interpolating the missing values using linear interpolation. The spike rate was then inferred from the fluorescence trace corrected from the motion artifacts using the OASIS deconvolution algorithm embedded in the package registration from suite2p, using a calcium decay of 1.8 s.

### Behavioral analysis and classification of swim bouts during high-speed volumetric imaging

#### Behavioral recording

Head-embedded larval zebrafish were illuminated from the side with a 45° angle using an 890 nm LED and the reflected image was recorded at 300 Hz from below through a ×5/0.25 12.5 mm microscope objective and imaged on a high-speed camera (UI-3060CP-M-GL R2, iDS) with a 50-mm lens. The 488-nm imaging laser was blocked with a 600-nm high-pass filter (Thorlabs) located in front of the camera. The high-speed camera was controlled using the Hiris software (RD Vision, https://www.rd-vision.com/r-d-vision-eng).

#### Tail tracking and bout extraction

We used the open-source software ZebraZoom (https://zebrazoom.org/) to track tail movement and extract bouts as discrete periods of time where motion was detected. We segmented the tail into 20 segments from the swim bladder to the tip of the tail and extracted the corresponding angles from each of those. For the tail curvature plots shown in Fig. 7, only the 17 most caudal segments were depicted. Proper tracking was assessed by visual inspection. The few swim bouts with clear tracking errors were discarded from the analysis.

#### Analysis of behavioral kinematics

Using ZebraZoom, we extracted different kinematic parameters to characterize swim bouts (https://zebrazoom.org/): absolute maximal bend amplitude, median bend amplitude, median TBF, bout duration and the number of oscillations. A bend corresponds to the maximum position of the tail tip on one side during a swim cycle. We computed the median TBF from the iTBFs calculated for each cycle as the inverse of the duration from the previous cycle. Median TBA was computed as the median value of the absolute value of the TBA during a swim bout.

#### Classification of swim bouts as forward, left or right turns

We classified forward swims and turns according to their maximum tail angle. Swim bouts whose maximum TBA was below 25° were classified as forward swims. Swim bouts whose maximum TBA was between 25 and 60° were classified as turns, and the sign was assigned according to the sign of their maximum tail angle. Swim bouts whose maximum TBA exceeded 60° were discarded from the analysis because most of them corresponded to escape or struggle-like behaviors.

We checked whether enucleated, head-embedded larval zebrafish exhibited forward and turn swim bouts that recall locomotor bouts typically exhibited by freely swimming larvae during exploration. To do so, we manually classified the swim bouts by applying a threshold (25°) on the tail angle. To exclude locomotor episodes that contained escape or struggle-like behavior that is not naturally exhibited by the larvae during exploration, we excluded all swim bouts with a maximum tail amplitude higher than 60°. We could successfully identify forward, left and right turns. At the level of a single experiment, the swim bouts classified as belonging to the same type were highly similar (Fig. 7d, top). We also noticed that, except for a characteristic initial bend of large amplitude on one side during the turn that steers the tail for the duration of the locomotor episode, the kinematics of tail curvature were strikingly similar between forward, left turns and right turns. In all cases, a wave of oscillations goes from the head to the tip of the tail of similar amplitude and propagation speed during forward, left and right turns (Fig. 7d, bottom).

### High-speed volumetric imaging experiments and neuronal recruitment

#### Enucleation procedure and its effect on locomotion

For enucleation, 3–4-dpf larvae were mounted in 3% agarose (Thermo Fisher Scientific) diluted in 1× sterile artificial cerebrospinal fluid. We used a sharp tungsten electrode bent into a hook shape that was cleaned before each procedure using ethanol 70%. The tool was gently inserted between the larva eye and orbit to cut the optic tract without damaging the surrounding tissue. The detached eye was then removed and the larvae were freed from agarose and kept in artificial cerebrospinal fluid for at least 45 min. Larvae were then placed back in Danieau’s solution 0.3×. On the day preceding the imaging experiments, 5-dpf larvae were placed in a 3.5-cm glass bottom Petri dish and embedded in 3% agarose. The agarose surrounding the tail, caudal to the swim bladder, was gently removed to leave the tail free to move. The dish was then filled with Danieau’s solution 0.3×. The larvae were imaged the day after the mounting procedure.

To estimate the effect of enucleation on locomotion, we compared the swimming kinematics of larvae with and without eyes during SCAPE recordings (Fig. 7). We found that enucleated larvae produced longer swim bouts with more oscillations. Moreover, enucleated larvae produced more symmetrical swims with lower TBA. Intact larvae swam faster than enucleated ones. On closer inspection, enucleated larvae exhibited a similar unimodal, non-Gaussian distribution of mean TBF centered around lower values than for intact siblings. Although the duration for enucleated larvae was longer, the proportion of time spent swimming was not significantly different between the two conditions.

#### In vivo recording of calcium imaging activity using the SCAPE microscope

For the in vivo recording, 6-dpf enucleated *Tg(vsx2:GAL4;UAS:GCaMP6s)* larvae were placed under the ×20 objective and placed at optimal *x*, *y* and *z* positions. The FOV was set to capture the fluorescence from the entire hindbrain and, when possible, the first segments of the spinal cord. For whole hindbrain recordings, the volume rate of acquisition was always set between 4 and 5.55 volumes per second, and the scanning step (*x*’ step) was set to 1.5 µm. The recordings lasted 10 min. Acquisition was synchronized to behavior monitoring by sending, using the MATLAB graphic user interface (MathWorks), a short TTL pulse to a waveform generator feeding frame signals to the high-speed camera. After the functional recording, we imaged the larval brain using a high-resolution stack with smaller steps of sampling to get an anatomical image for future registration to a zebrafish brain atlas.

#### Calcium imaging data analysis and statistical tests

##### Detection of active neurons during swim bouts

To detect if a given neuron was active during a type of swim bouts (forward, left turns, right turns), we compared the distribution of spikes during all swim bouts of a given type to the distribution of inferred spike rates when the fish was not swimming. To build this distribution for a given neuron and a given type of swim bout, we processed as follows: (1) for a given swim bout, we extracted the spiking rates between 200 ms before the start of the bout and the end of the bout; (2) we compiled these spiking rates for all swim bouts of the same type; (3) we sampled the spiking rate when the fish was not moving as periods of time where the tail angle was zero; (4) these distributions did not follow a Gaussian distribution and did not have the same sample size. Therefore, we used a nonparametric Wilcoxon rank-sum test to test if the distribution of spiking rate during the given type of bouts was greater than the distribution of spiking rate when the fish was not swimming. We used a threshold *P* = 0.01 to reject the null hypothesis. Thus, the neuron was labeled as active during the type of swim bout if the resulting *P* value from the test was lower than 0.01.

##### Detection of forward component and steering neurons

Using the method stated above, we extracted all neurons active during each bout type as cells whose spiking rate during each bout of the same type (forward bout, left turn or right turn) was significantly higher than the distribution of spiking rate during the immotile period. We then computed two groups of interest as follow: forward-component neurons: neurons active during both forward, left and right turns; left steering neurons: neurons active during left turns, but not during forward; right steering neurons: neurons active during right turns, but not during forward. Of 11 enucleated larvae recorded, we kept for this analysis three larvae that produced at least 50 swim bouts and at least one bout of each category (forward, left turn, right turn).

### Data visualization and statistical analysis

Analysis was done either in R (v.4.0.4) or Python v.3.7. Initial figures were done either in R using the tidyverse package (v.1.3.0) or using the matplotlib (v.3.3.2) and seaborn (v.0.11.0) Python libraries. Figures were assembled in Inkscape (v.1.1, https://inkscape.org/). All statistical tests applied are specified in the legend of the corresponding figure. All data met the assumptions of the statistical tests used. Data collection and analysis were not performed blind to the conditions of the experiments except for the manual segmentation and categorization of behavior in Figs. 2 and 3. Statistical tests were performed in R, except for the statistical analysis in Fig. 7 (details in the previous section), and the multiple linear models used in Fig. 8, for which we used the OLS function from the library stats.models developed for Python (https://github.com/statsmodels/statsmodels).

### Reporting summary

Further information on research design is available in the Nature Portfolio Reporting Summary linked to this article.

## Online content

Any methods, additional references, Nature Portfolio reporting summaries, source data, extended data, supplementary information, acknowledgements, peer review information; details of author contributions and competing interests; and statements of data and code availability are available at 10.1038/s41593-023-01418-0.

### Supplementary information

Reporting Summary

Supplementary Video 1 Electrical stimulation in the MLR locus only elicits sustained forward swimming. Tail beats of a head-embedded larva were monitored at 300 frames per second during the 4-s-long electrical stimulation. Playback has been slowed down 4×.

Supplementary Video 2 The axon of an MLR neuron reaching the soma of a pontine RSN. Z-stack showing the axon of an MLR neuron (orange, top) reaching the soma of a V2a pontine RSN (‘RoM2’, cyan, bottom). Overlap is indicated in white.

Supplementary Video 3 Medullary V2a RSNs show an increase in intracellular calcium during MLR stimulation-induced forward swimming. A 40-s-long MLR stimulation induces exceptionally long forward swims that can be intermingled with struggle and escape-like behaviors. Stimulation of the MLR recruits medullary V2a RSNs during the sustained forward swims.

### Source data

Source Data Fig. 2 Microsoft Excel worksheet for panels c,d,f,l,n.

Source Data Fig. 3 Data frame for panels b,c,d,f,g,h,n.

Source Data Fig. 4 Data frame for panels f and g.

Source Data Fig. 5 Data frame for panels c–f.

Source Data Fig. 6 Images for panels a,d,e,f,g.

Source Data Fig. 7 Data frame for panels c,d,g,h,i.

Source Data Fig. 8 Data frame for panels b,g,h,j,k.

Source Data Extended Data Fig. 2 Data frame.

Source Data Extended Data Fig. 4 Data frame.

## Data Availability

Source data are available as supplementary information. Source data are provided with this paper.

## Code availability

All Python scripts used to process the data are available at https://github.com/wyartlab/Carbo-Tano_Lapoix_2023 and https://github.com/mpascucci/pycroscopy3D.
