# Supplementary figures and images for: The mesencephalic locomotor region recruits V2a reticulospinal neurons to drive forward locomotion in larval zebrafish
Source: Nat Neurosci. 2023 Sep 4;26(10):1775–90. doi: 10.1038/s41593-023-01418-0 (PMC10545542; doi:10.1038/s41593-023-01418-0)

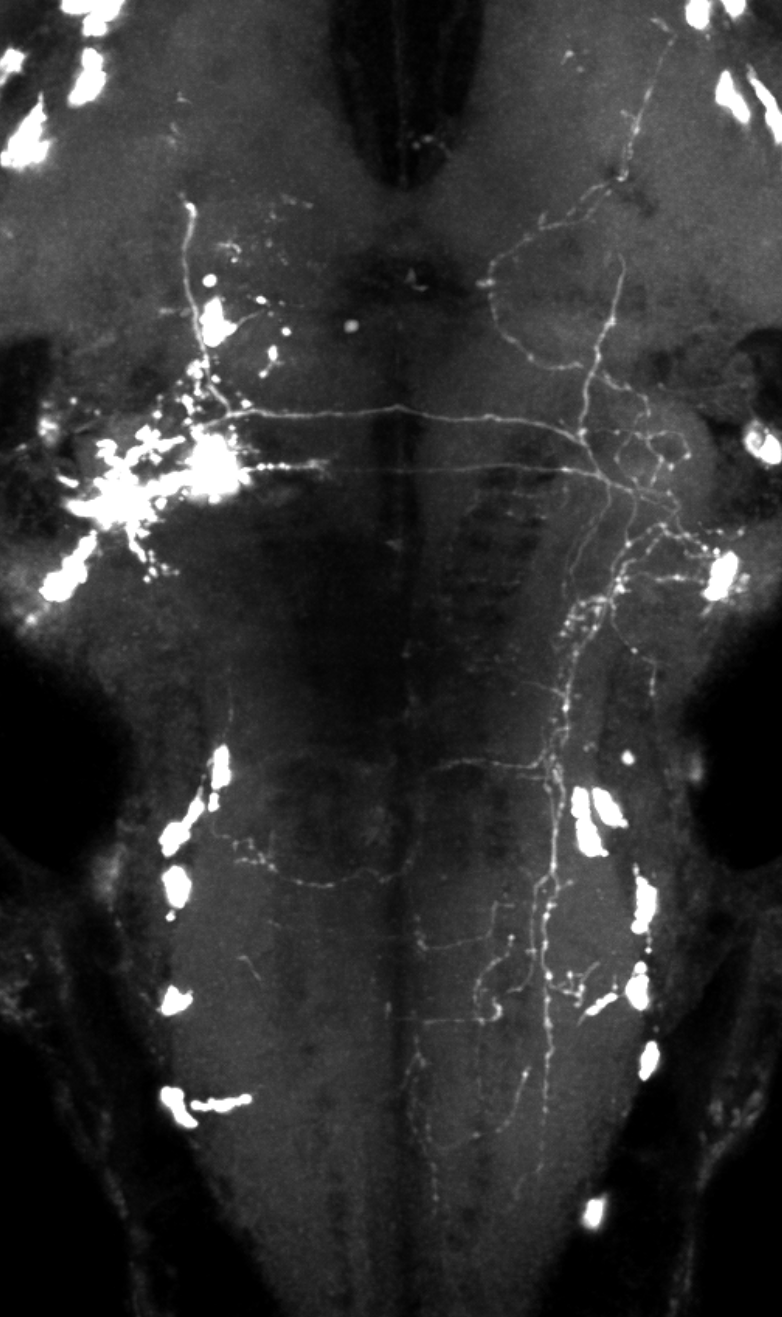

Supplement: Supplementary file 9 — Images for panels a,d,e,f,g. [file 41593_2023_1418_MOESM9_ESM.zip › Fig6a.tiff]

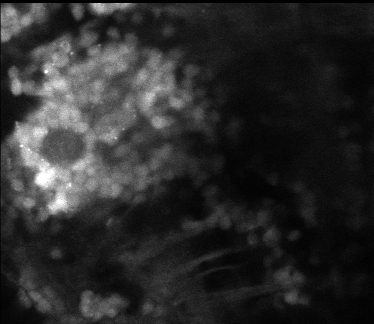

Supplement: Supplementary file 9 — Images for panels a,d,e,f,g. [file 41593_2023_1418_MOESM9_ESM.zip › Fig6e.tif]
